# Cyclic nucleotides in archaea: Cyclic di‐AMP in the archaeon *Haloferax volcanii *and its putative role

**DOI:** 10.1002/mbo3.829

**Published:** 2019-03-18

**Authors:** Frank Braun, Laura Thomalla, Chris van der Does, Tessa E. F. Quax, Thorsten Allers, Volkhard Kaever, Sonja‐Verena Albers

**Affiliations:** ^1^ Molecular Biology of Archaea Institute of Biology, University of Freiburg Freiburg Germany; ^2^ School of Life Sciences, Queen's Medical Centre University of Nottingham Nottingham UK; ^3^ Research Core Unit Metabolomics, Hannover Medical School Hannover Germany

**Keywords:** archaea, cyclic di‐AMP, diadenylate cyclase, *Haloferax volcanii*, osmoregulation, second messenger

## Abstract

The role of cyclic nucleotides as second messengers for intracellular signal transduction has been well described in bacteria. One recently discovered bacterial second messenger is cyclic di‐adenylate monophosphate (c‐di‐AMP), which has been demonstrated to be essential in bacteria. Compared to bacteria, significantly less is known about second messengers in archaea. This study presents the first evidence of in vivo presence of c‐di‐AMP in an archaeon. The model organism *Haloferax volcanii* was demonstrated to produce c‐di‐AMP. Its genome encodes one diadenylate cyclase (DacZ) which was shown to produce c‐di‐AMP in vitro. Similar to bacteria, the *dacZ* gene is essential and homologous overexpression of DacZ leads to cell death, suggesting the need for tight regulation of c‐di‐AMP levels. Such tight regulation often indicates the control of important regulatory processes. A central target of c‐di‐AMP signaling in bacteria is cellular osmohomeostasis. The results presented here suggest a comparable function in *H. volcanii*. A strain with decreased c‐di‐AMP levels exhibited an increased cell area in hypo‐salt medium, implying impaired osmoregulation. In summary, this study expands the field of research on c‐di‐AMP and its physiological function to archaea and indicates that osmoregulation is likely to be a common function of c‐di‐AMP in bacteria and archaea.

## 1 INTRODUCTION

Bacteria use cyclic nucleotide derivates to relay environmental signals to downstream receptors and thereby modulate various cellular pathways in response to changes in their environment (Gomelsky, 2011; Kalia et al., 2013; McDonough & Rodriguez, 2011). Therefore, these molecules are mostly referred to as second messengers. One such second messenger that is conserved in many bacterial species is cyclic di‐adenylate monophosphate (c‐di‐AMP) (Römling, 2008; Witte, Hartung, Büttner, & Hopfner, 2008). It is involved in various cellular processes and pathways such as DNA integrity sensing (Bejerano‐Sagie et al., 2006; Witte et al., 2008), control of cell size and cell wall homeostasis (Corrigan, Abbott, Burhenne, Kaever, & Gründling, 2011), regulation of fatty acid synthesis (Zhang, Li, & He, 2013), and maintenance of the osmotic homeostasis of the cell (Bai et al., 2014; Corrigan et al., 2013; Gundlach et al., 2017). Based on current knowledge, eukaryotic cells do not use c‐di‐AMP as second messenger. However, c‐di‐AMP‐based signaling is not completely absent in eukaryotes. It has been reported that c‐di‐AMP released by intracellular pathogens, such as *Listeria monocytogenes* and *Mycobacterium tuberculosis*, activates the hosts' immune system by triggering a type I interferon response (Woodward, Iavarone, & Portnoy, 2010; Yang et al., 2014). Amongst cyclic nucleotide‐based second messengers, c‐di‐AMP has a unique position as it is (under standard growth conditions) the only second messenger that is essential for the vast majority of species that produce it (Blötz et al., 2017; Chaudhuri et al., 2009; Luo & Helmann, 2012; Song et al., 2005; Whiteley, Pollock, & Portnoy, 2015). In addition, the intracellular level of c‐di‐AMP needs to be tightly regulated as both decreased and increased amounts of c‐di‐AMP in the cytoplasm cause severe negative effects on the cells (Gundlach et al., 2015; Huynh & Woodward, 2016).

In vivo, c‐di‐AMP is synthesized from two molecules of adenosine triphosphate (ATP) by diadenylate cyclases (DACs) (Witte et al., 2008), and is degraded to two molecules of adenosine monophosphate (AMP) by phosphodiesterases (PDEs) (Manikandan et al., 2014). Phylogenetic analysis has revealed several classes of DACs, all of which contain a conserved DAC domain (also called DisA_N domain [Pfam: PF02457]) in addition to several other functionally different domains (Commichau, Heidemann, Ficner, & Stülke, 2018; Corrigan & Gründling, 2013). Hypothetical proteins containing predicted DAC domains are present in diverse bacterial but also some archaeal species of the phylum Euryarchaeota, in which a previous study identified five unique putative DAC classes (DacV–DacZ) (Corrigan & Gründling, 2013). For the archaeal DacY class (also referred to as CdaZ class (Commichau et al., 2018)) it has been shown that a representative of this class, CdaZ from *Methanocaldococcus jannaschii*, exhibits DAC activity when expressed in a heterologous host (Commichau et al., 2018; Kellenberger, Chen, Whiteley, Portnoy, & Hammond, 2015). However, the actual production of c‐di‐AMP in vivo has not been reported for any archaeal species so far.

Research on cyclic nucleotides started with the discovery of cyclic AMP (cAMP) in 1956 (Sutherland & Wosilait, 1956). Since then, various cyclic nucleotide second messengers and their corresponding signaling pathways have been revealed in bacteria and eukaryotes. However, surprisingly little is known about the presence and molecular function of cyclic nucleotides in archaea. In 1986, the presence of cAMP was reported in the archaeal species *Methanobacterium thermautotrophicus*, *Sulfolobus solfataricus*, and *Haloferax volcanii* (then referred to as *Halobacterium volcanii*) (Leichtling, Rickenberg, Seely, Fahrney, & Pace, 1986), and cAMP levels have also been investigated in *Halobacterium salinarum* where a cell cycle‐dependent fluctuation of the intracellular cAMP concentration has been observed (Baumann, Lange, & Soppa, 2007). Another cyclic nucleotide that has been characterized in archaea is cyclic oligoadenylate, which has been shown to be involved in type III CRISPR‐mediated immunity in *S. solfataricus* (Rouillon, Athukoralage, Graham, Grüschow, & White, 2018). Comparative genome analyses have predicted different cyclases, including DACs, in additional archaeal species (Corrigan & Gründling, 2013; Shenroy & Visweswariah, 2004). However, the above three mentioned studies represent the only direct in vivo evidence on any cyclic nucleotides in archaea to date.

In this study, we show that c‐di‐AMP is produced by the euryarchaeon *H. volcanii*, which is the first direct evidence on the in vivo presence of this cyclic nucleotide in an archaeal species. The genome of *H. volcanii* encodes a single DAC belonging to the proposed DacZ subfamily (Corrigan & Gründling, 2013). The *dacZ* gene is constantly expressed at different growth phases in rich and selective media. Additionally, the gene is essential indicating that c‐di‐AMP is essential for *H. volcanii*, as reported for various bacteria. Overexpression of DacZ caused cell death suggesting the need for tight regulation of intracellular c‐di‐AMP levels. Data on in vitro enzymatic activity of heterologously expressed DacZ confirmed its identity as DAC. To investigate the function of c‐di‐AMP‐related signaling in *H. volcanii*, the expression of *dacZ* in media containing low sodium chloride concentrations was modulated using two different tryptophan‐inducible promoters; this revealed a possible link between intracellular c‐di‐AMP levels and osmohomeostasis.

## 2 MATERIAL AND METHODS

Unless stated otherwise all chemicals were purchased from Carl Roth.

### 2.1 Strains and growth conditions

*Haloferax volcanii* strains were either grown in rich YPC (Allers, Ngo, Mevarech, & Lloyd, 2004) (0.5 g/L Yeast Extract [Oxoid]; 0.1 g/L Peptone [Oxoid]; 0.1 g/L Bacto™ Casamino acids [BD biosciences]; pH 7.2 adjusted with KOH) medium or selective CA (Allers et al., 2004) (0.5 g/L Bacto™ Casamino acids; pH 7.2 adjusted with KOH) medium modified with an expanded trace element solution (referred to as CAB) (Duggin et al., 2015). Strains were grown at 45°C in liquid medium rotating (volumes up to 5 ml) or shaking at 170 rpm (volumes > 5 ml). Strains with auxotrophic marker deletions were grown in CAB medium supplemented with the respective compound (50 µg/ml uracil [Sigma] for Δ*pyrE2*; 50 µg/ml tryptophan for Δ*trpA*; 40 µg/ml thymidine & hypoxanthine [Sigma] for Δ*hdrB*).

For CAB media with defined NaCl concentrations (concentrations of other components were not altered) used in salt stress experiments (1.8 M: hypo‐salt; 2.5 M: standard‐salt; 4.5 M: high‐salt), media without NaCl and with 5 M NaCl were mixed in defined proportions to obtain media with the desired final NaCl concentration.

*Escherichia coli* strains were cultivated in Lysogeny Broth‐medium (LB‐medium Luria/Miller) (Miller, 1971) supplemented with antibiotics if necessary (100 µg/ml ampicillin; 25 µg/ml kanamycin; 30 µg/ml chlorampenicol) at 37°C under constant shaking at 170 rpm.

All strains used in this study are listed in Table A1.

### 2.2 Plasmid construction

Plasmids transformed into *H. volcanii* were cloned using classical restriction enzyme‐based molecular cloning. Inserts were amplified from wild type (H26) genomic DNA (isolated as described previously (Allers et al., 2004)) via polymerase chain reaction (PCR) using PHUSION® polymerase (NEB). All restriction endonucleases used were purchased from NEB. Restriction enzyme digestions and PCR were performed according to the manufacturer’s protocols. All plasmids transformed to *H. volcanii* were isolated from *dam^−^*/*dcm^−^* competent *E. coli* cells (NEB).

Plasmids for heterologous protein overexpression in *E. coli* were cloned using FX‐cloning system as described earlier (Geertsma & Dutzler, 2011). Primers, backbones, and restriction enzymes used are summarized in Tables A2 and A3.

### 2.3 Genetic manipulation of H. volcanii and creation of deletion mutants

Transformation and construction of deletion mutants in *H. volcanii* H26 based on selection with uracil were performed as described previously (Allers et al., 2004). In brief: pTA131‐based deletion constructs, carrying a *pyrE2*‐cassette and the desired knock‐out construct consisting out of ~500 bp upstream and downstream flanking regions of the target gene, were transformed to the desired background strain. Transformed cells were plated on selective CA plates and incubated for 5 days. Single pop‐in colonies were restreaked on CA plates and, following a 3‐day incubation, cell material was inoculated in 5 ml‐rich YPC medium for unselective growth to relieve selective pressure for the *pyrE2* cassette on the integrated plasmid. Nonselective growth in YPC was repeated three times o/n. Selection for pop‐out cells was achieved by plating on CA medium containing 50 µg/ml 5‐fluoroorotic acid (5‐FOA) (Fermentas) and 10 µg/ml uracil. After incubation for 5 days, up to 100 cells were transferred to a YPC plate and incubated for 2 days. These colonies were subjected to a colony blot hybridization on a Zeta®‐Probe GT blotting membrane (BioRad). DNA was UV cross‐linked to the membrane at 120 mJ/cm^2^. Subsequently, the membrane was subjected to pre‐hybridization and hybridization using the DIG (Digoxigenin) High Prime DNA labelling and detection starter kit II (Roche) according to the manufacturer's protocol. Colonies which did not hybridize to the probe were grown in 5 ml YPC for isolation of genomic DNA. Gene deletions were validated via PCR analysis using primers annealing outside of the flanking regions used in the knock‐out plasmid. PCR products of putative deletion strains were compared to corresponding PCR products of the wild type strain H26 and verified through sequencing.

Creation of mutants with an exchange of the native promotor of *dacZ* with the tryptophan‐inducible promotor (p.*tnaA*) (Large et al., 2007) used an integrative approach described previously (Skowyra & MacNeill, 2012). Briefly, the coding sequence of *dacZ* was cloned in pTA1369 (induction with p.*tnaA*)/pTA1451 (induction with p.*tna*M3); pTA1369 and pTA1451 are shown in Figure A11. The resulting inducible gene +hdrB marker construct was digested with *Bgl*II and inserted at the *BamH*I site located between the upstream and downstream flanking regions of *dacZ* in the *dacZ* gene deletion construct (pSVA3930). Transformation and pop‐in procedure in H98 were performed as described for gene deletion trials. Correct upstream pop‐in position and orientation were examined using PCR. Pop‐out procedure was performed as described above with maintained selection for *hdrB*. 5‐FOA^R^ colonies were checked for promotor exchange by PCR and sequencing.

Verification of *dacZ*s essentiality based on a *in trans* complementation approach reported previously (Lestini, Duan, & Allers, 2010). In brief: The triple auxotroph strain H99 (Δ*pyrE2*; Δ*trpA*; Δ*hdrB*) was transformed with a *trpA*‐marked *dacZ* deletion construct (pSVA5420). The resulting pop‐in strain HTQ409 was transformed with the episomal plasmids pSVA5421 (pTA356 + *dacZ*), pSVA5422 (pTA409 + *dacZ*), and corresponding empty backbone controls. The pop‐out procedure for pSVA5421 and pTA356 (Norais et al., 2007) was performed in medium without thymidine. Pop‐outs for pSVA5422 and pTA409 (Delmas, Shunburne, Ngo, & Allers, 2009) in medium with thymidine (releasing selection pressure for *hdrB*). For each construct, 5‐FOA^R^ colonies were checked for complete deletion of *dacZ* from its chromosomal locus by PCR and sequencing. Additionally, complete deletion was confirmed by specific restriction digest (using *Stu*I + EcoRV) and Southern Blotting. To demonstrate essentiality of *dacZ*, the genomic *dacZ* deletion strain carrying pSVA5421 (HTQ413) was grown three times overnight in liquid CA + uracil + thymidine & hypoxanthine followed by two consecutive rounds of growth on nonselective CA + uracil + thymidine & hypoxanthine plates to allow loss of pSVA5421. As a control for plasmid loss, H98 (Δ*pyrE2*; Δ*hdrB*) was also transformed with pSVA5421 and subjected to identical unselective growth conditions. Loss of plasmid was checked by transferring nonselectively grown colonies to selective plates without thymidine & hypoxanthine.

### 2.4 Phase‐contrast light microscopy and cell shape quantification

*Haloferax volcanii* cells were imaged at a magnification of 1000x using an Axio Observer.Z1 inverted microscope (Zeiss). For imaging, cell cultures were diluted back to an OD_600_ of 0.1 and 3 µl of the diluted cell suspension was spotted on an agarose pad (0.4% [w/v] agarose in 18% salt water [144 g/L NaCl; 21 g/L MgSO_4_ * 7 H_2_O; 18 g/L MgCl_2_ * 6 H_2_O; 4.2 g/L KCl; 12 mmol/L Tris/HCl, pH 7.5]), and allowed to air dry. The agarose pad was covered with a coverslip and visualized. For each strain, at least 500 cells/biological replicate were used for cell morphology evaluation. Evaluation of cell shape was performed using Fiji (Schindelin et al., 2012) in combination with the MicrobeJ plug‐in (Ducret, Quardokus, & Brun, 2016).

### 2.5 RNA isolation and dacZ expression level determination by quantitative PCR

RNA was isolated from *H. volcanii* H26 cells growing exponentially/stationary in YPC/CAB using the RNeasy® Mini Kit (Qiagen) in combination with the RNAse‐Free DNAse Set (Qiagen) for on column genomic DNA digestion according to the manufacturer's protocol. cDNA synthesis was performed using the QuantiNova™ Reverse Transcription Kit (Qiagen) according to the manufacturer's protocol with optimized duration of the reverse transcription step of 13 min. Analysis of cDNA libraries was performed with a final assay volume of 10 µl/sample using the qPCRBIO SyGreen Mix Lo‐ROX (PCRBIOSYSTES) as advised by the manufacturer. Quantitative PCR (qPCR) cycling was conducted in a Magnetic Induction Cycler (MIC) (Bio Molecular Systems) with the following cycling parameters: 2 min initial denaturation at 95°C; 40 cycles with 5 s denaturation at 95°C, and 30 s elongation at 65°C. For absolute quantification of *dacZ* transcripts, a standard curve was created using a 1,029 bp *dacZ* PCR product at defined concentrations as template. Absolute expression of *dacZ* in YPC‐stationary, CAB‐exponential, and CAB‐stationary phase was normalized to expression in YPC‐exponential phase.

### 2.6 c‐di‐AMP extraction from H. volcanii cells

The extraction of c‐di‐AMP from *H. volcanii* cells was performed as described previously (Spangler, Böhm, Jenal, Seifert, & Kaever, 2010). Briefly, *H. volcanii* cells were grown in 340 ml YPC/CAB or 50 ml CAB with 1.8 M NaCl. For exponentially grown cells 20 ml, for stationary grown cells 15 ml were harvested. For each time point, an additional 2 ml aliquot of each culture was harvested for the determination of total protein content (BCA Protein Assay Macro Kit [Serva]). Cell pellets were snap‐frozen in liquid nitrogen and subsequently resuspended in 300 µl extraction solution (acetonitrile/methanol/water: 2:2:1 [v/v/v]). Resuspended pellets were incubated in ice for 15 min followed by a heating step at 95°C for 10 min. After cooling on ice, the solution was centrifuged for 10 min at 4°C and maximum speed (21,100× *g*). The resulting supernatant was transferred to a fresh vial. The extraction was repeated two times (three extraction steps in total) with 200 µl fresh extraction solution, omitting the heating step. Combined supernatants were stored overnight at −20°C to precipitate protein. To remove precipitates, the samples were centrifuged again (10 min, 4°C; 21,100× *g*) and the supernatant was transferred to a fresh vial. Final extracts were desiccated using a vacuum concentrator (Eppendorf) at 45°C.

### 2.7 Quantification of c‐di‐AMP from cell extracts by liquid chromatography tandem mass spectrometry

Dried nucleotide extracts/enzyme assay mixtures were resuspended in 200 µl water, centrifuged and, following the addition of the internal standard ([^13^C,^15^N]c‐di‐AMP), analyzed by a liquid chromatography tandem mass spectrometry (LC‐MS/MS) method as described earlier (Mehne et al., 2013). The measured concentration of c‐di‐AMP was normalized for each cell extract sample to the total protein concentration of the respective sample.

### 2.8 Heterologous expression and purification of DacZ

Overexpression of *N*‐terminal 10x histidine‐tagged DacZ was performed by transforming pSVA5408 to chemically competent *E. coli* Rosetta (Novagen) cells. For expression, a preculture of transformed cells (grown in LB_Kana/Cam_) was diluted back to an OD_600_ of ~0.05 in 2 L of LB/SMM_Kana/Cam_ medium (conventional LB‐medium with osmotically active substances: 1x LB; 0.5 M sucrose; 16.85 mmol/L maleic acid; 50 mmol/L KCl; 20 mmol/L MgCl_2_; pH: 7.0) and grown to an OD_600_ of ~0.5. Induction was performed with 0.2 mmol/L isopropyl β‐D‐1‐thiogalactopyranoside (IPTG) (Fisher Science) for 3 hr at 37°C. Following harvesting by centrifugation, cell pellets were resuspended in *H. volcanii* protein purification buffer (1.5 M KCl; 100 mmol/L NaCl; 100 mmol/L HEPES; 1 mmol/L DTT; pH 7.5) supplemented with 10 µg/ml DNAseI (Roche). Cell lysis was accomplished by sonification followed by the removal of cell debris by centrifugation at 48,200× *g* at 4°C for 20 min. Supernatant was loaded on a 5 ml HisTrap™HP column (GE Healthcare) connected to an ÄKTApurifier fast protein liquid chromatography (FPLC) system (GE Healthcare) and eluted using *H. volcanii* protein purification buffer supplemented with 400 mmol/L imidazole. Elution fractions were checked for DacZ content by SDS‐PAGE and Western Blot. Imidazole was removed by dialysis overnight in 5 L of *H. volcanii* protein purification buffer (supplemented with 2.5 M EDTA) using a MEMBRA‐CEL® dialysis tubing with a molecular weight cutoff of 7.5 kDa (Serva). Following dialysis, the elution fractions were concentrated with a Microsep™ Advance Centrifugal Devices (Pall Corporation) with a 10 kDa cutoff. The final protein content of concentrated elution fractions was determined using the BCA (bicinchoninic acid) protein assay macro kit (Serva) according to the manufacturer’s protocol.

### 2.9 In vitro* activity determination of DacZ by thin‐layer chromatography using [α‐^32^P]ATP*

In vitro DAC activity assays and detection of radioactive c‐di‐AMP were adapted from Bai et al (Bai et al., 2012). DacZ activity assays were performed in a total volume of 25 µl in *H. volcanii* protein purification buffer supplemented with 10 mmol/L of MnCl_2_ (respectively 10 mmol/L of MgCl_2_; CaCl_2_; NiCl_2_; CoCl_2_ for the cofactor dependence experiment). Final protein concentration of purified DacZ within the assay mixture was 10 µmol/L. As substrate, 100 µmol/L unlabeled ATP (Sigma) (solved in 500 mmol/L Tris/HCl, pH 7.5) mixed with [α‐^32^P]ATP (Hartmann Analytic) to a final activity of 200 Ci/mmol (final concentration of [α‐^32^P]ATP: 55 nmol/L) was used. For testing activity with GTP, 100 µmol/L of unlabeled GTP (Sigma) (dissolved in 500 mmol/L Tris/HCl, pH 7.5) mixed with [α‐^32^P]GTP (Hartmann Analytic) to a final activity of 167 Ci/mmol (final concentration of [α‐^32^P]GTP: 66 nmol/L) was used. Reactions were started by the addition of ATP. Assays were incubated at 37°C for 1 hr and stopped by adding an equal volume of 0.5 M EDTA. One microliter of this mixture was spotted on Polygram® CEL 300 polyethyleneimine‐cellulose thin‐layer chromatography (TLC) plates (Machery‐Nagel) and allowed to air dry. Plates were developed in 1:1.5 (v/v) saturated NH_4_SO_4_ and 1.5 M KH_2_PO_4_, pH 3.6, air dried and exposed to a phosphor imaging screen BAS‐MS 2040 (Fujifilm) overnight. Imaging plates were developed using a Typhoon FLA 9500 (GE Healthcare).

### 2.10 In vitro* activity of DacZ and product verification by LC‐MS/MS*

To verify c‐di‐AMP as in vitro, DacZ product assays were performed using an identical setup as described in the preceding section. However, the total volume of the assay was increased to 350 µl and 100 µmol/L ATP was added. The assay was incubated for 1 hr at 37°C. Following incubation, the reaction was stopped by heating the assay mixture at 95°C for 5 min. The inactivated assay mixture was mixed with 1.4 ml assay extraction solution (acetonitrile/methanol: 1:1 [v/v]) and incubated overnight at −20°C. The mixture was then centrifuged for 10 min at 4°C and maximum speed (21,100x *g*). The resulting supernatant was transferred to a fresh vial and evaporated to dryness using a vacuum concentrator (Eppendorf) at 45°C.

## 3 RESULTS

### 3.1 The genome of H. volcanii encodes for one putative DAC belonging to the DacZ/CdaZ subfamily

**Figure 1:**
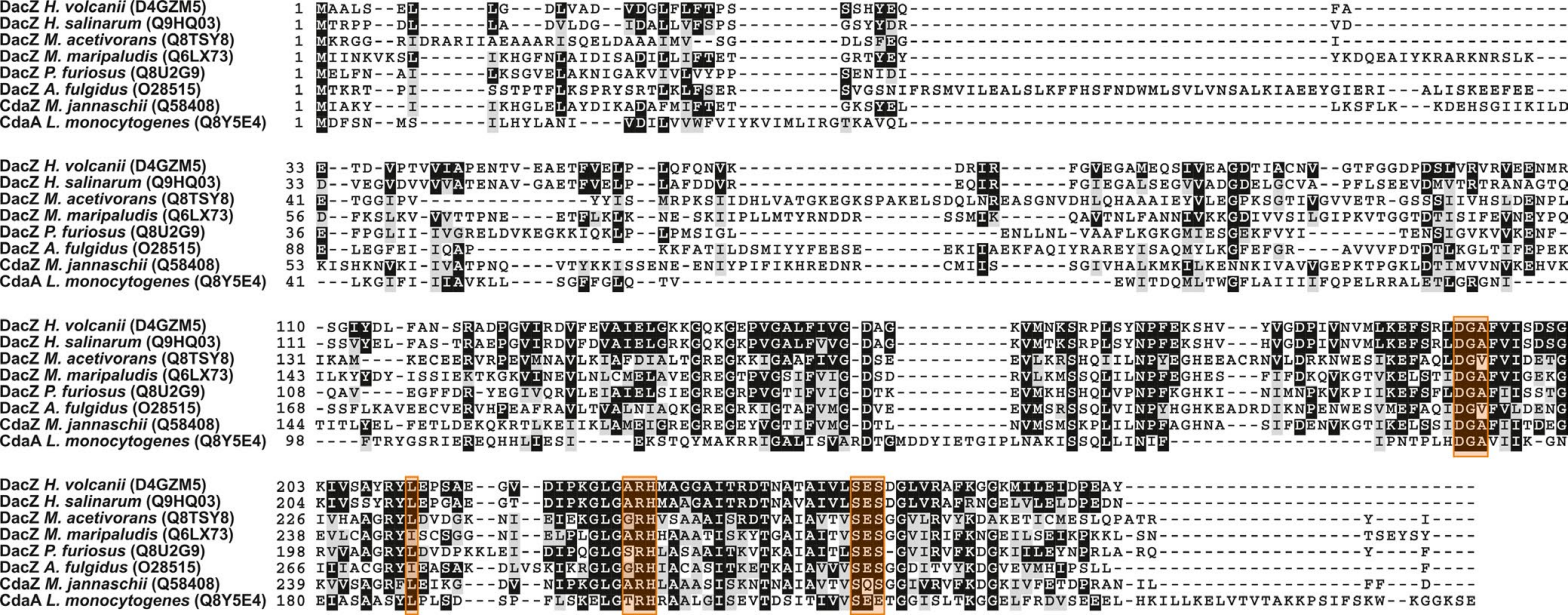
Alignment of euryarchaeal diadenylate cyclase DacZ proteins (protein ID in brackets) with the biochemically and structurally characterized bacterial DAC CdaA from *Listeria monocytogenes* (Rosenberg et al., 2015). Conservation of catalytically important residues within the DAC domain (Rosenberg et al., 2015) is indicated by orange rectangles

**Figure A1:**
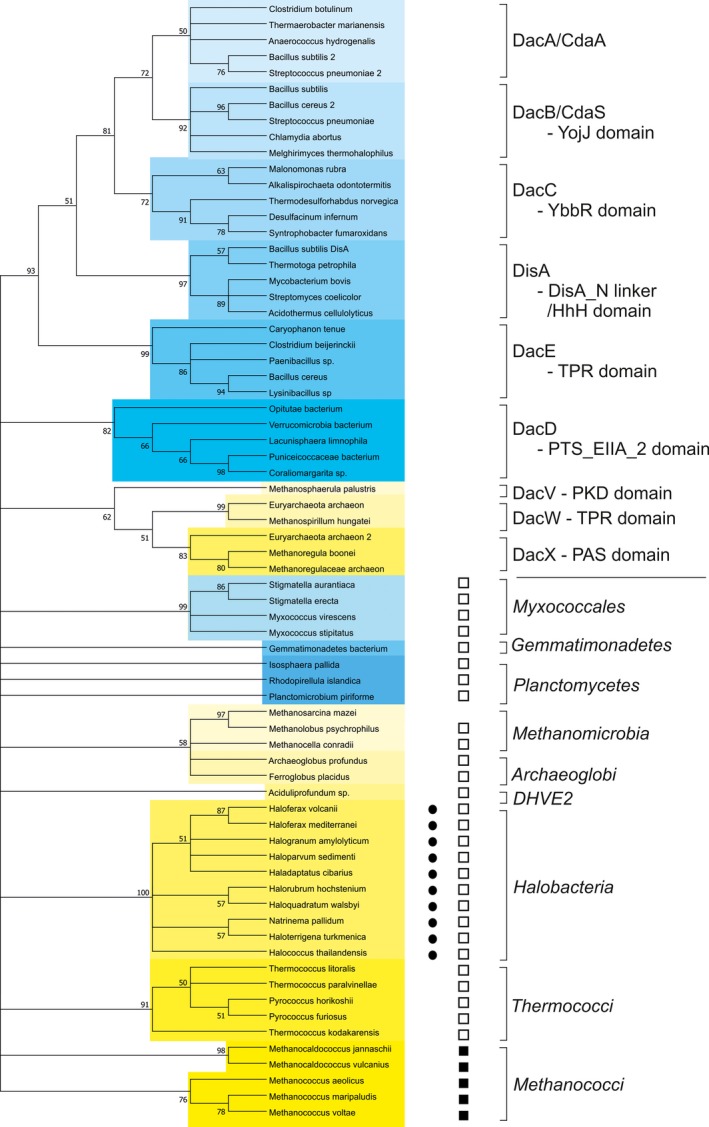
Molecular Phylogenetic analysis of *Haloferax volcanii* diadenylate cyclase (DacZ) and other di‐adenylate cyclases by Maximum Likelihood method. To analyze the phylogenetic relation between *H. volcanii* DacZ and other di‐adenylate cyclases, sequences related to DacY/CdaZ and representatives from previously identified bacterial and archaeal classed were collected (Commichau et al., 2018; Corrigan & Gründling, 2013), the adenylate cyclase domains were aligned, all positions containing gaps and missing data were eliminated and a phylogenetic tree was constructed (for details see below) using the Maximum Likelihood method based on the JTT matrix‐based model (Jones, Taylor, & Thornton, 1992). A bootstrap consensus tree inferred from 500 replicates is shown. Branches corresponding to partitions reproduced in less than 50% bootstrap replicates are collapsed. The percentage of replicate trees in which the associated taxa clustered together in the bootstrap test are shown next to the branches (Felsenstein, 1985). There were a total of 99 positions in the final dataset. Evolutionary analyses were conducted in MEGA7 (Kumar, Stecher, & Tamura, 2016). Within the tree, bacteria are indicated in blue, while archaea are indicated in yellow. On the right side, either the corresponding families of adenylate cycles are indicated (upper part), or the order or class to which the organisms belong is indicated (lower part). The closed circles indicate proteins which are encoded in an operon with a gene encoding a MscS channel. Closed black squares indicate proteins where a *N*‐terminal PK_C pyruvate kinase C‐terminal alpha/beta domain was identified via a conserved domain search (Marchler‐Bauer et al., 2017; result mode: Full). Open squares indicate proteins for which the Swiss‐model server (https://swissmodel.expasy.org) (Waterhouse et al., 2018) predicts an *N*‐terminal PK_C pyruvate kinase C‐terminal alpha/beta domain. Detailed method: *H. volcanii* DacZ was blasted against the NCBI non redundant database and the first 1,000 hits were selected and sequences with a similarity above 99% were removed, resulting in 600 sequences. Then, 10 representatives of the bacterial DacA/CdaA, DacB/CdaS, DacC, DisA, DacD classes, six representatives of the bacterial DacE, and 1, 2, 3, 10 and 10 representatives of the archaeal DacV, DacW, DacX, DacY/CdaZ and DacZ classes were selected. These sequences were combined and aligned using the MUSCLE algorithm. *N*‐ and C‐terminal sequences that did not encode for the di‐adenylate cyclase domain were removed, sequences were aligned again, and an initial phylogenetic tree was inferred by using the Maximum Likelihood method based on the JTT matrix‐based model (Jones et al., 1992). The bootstrap consensus tree inferred from 500 replicates (Felsenstein, 1985) was taken to represent the evolutionary history of the taxa analyzed (Felsenstein, 1985) Five sequences were selected that were representative of the main branches representing the DacA/CdaA (*Bacillus subtilis* [CDAA_BACSU], *Clostridium botulinum* [C5UT34_CLOBO], *Anaerococcus hydrogenalis* [A0A133LPS3_9FIRM], *Streptococcus pneumoniae* [A0A0B7L730_STREE], *Thermaerobacter marianensis* [013494666.1]), DacB/CdaS (*B. subtilis* [CDAS_BACSU], *Bacillus cereus* [J8JE08_BACCE], *Chlamydia abortus* [A0A1X6W494_CHLAO], *S. pneumoniae* [A0A0U0GIF0_STREE], *Melghirimyces thermohalophilus* [A0A1G6LWF9_9BACL]), DacC (*Desulfacinum infernum* (A0A1M5FM14_9DELT), *Alkalispirochaeta odontotermitis* (A0A0A0DNA5_9SPIO), *Syntrophobacter fumaroxidans* (A0LNX4_SYNFM), *Malonomonas rubra* (A0A1M6KRJ1_MALRU), *Thermodesulforhabdus norvegica* (A0A1I4QRW4_9DELT), DacD (*Lacunisphaera limnophila* (A0A1D8AXP5_9BACT), *Puniceicoccaceae bacterium* (A0A2A5VW89_9BACT), *Coraliomargarita *sp. (A0A1Z8WJT9_9BACT), *Verrucomicrobia bacterium* (A0A1Q6UHC2_9BACT), *Opitutae bacterium* (A0A2D5Y9T6_9BACT), DacE (*Caryophanon tenue* [A0A1C0YAV7_9BACL], *B. cereus* [A0A2C1SCY1_BACCE], *Lysinibacillus *sp.* A *[0A1T5G8C8_9BACI], *Clostridium beijerinckii* [A0A0B5QT99_CLOBE], *Paenibacillus *sp. [A0A198A0N3_9BACL]) and DisA (*B. subtilis* [DISA_BACSU], *Streptomyces coelicolor* [DISA_STRCO], *Acidothermus cellulolyticus* [DISA_ACIC1], *Thermotoga petrophila* [DISA_THEP1], *Mycobacterium bovis* [DISA_MYCBO]) classes. Also, representatives for the archaeal classes, DacV (*Methanosphaerula palustris* [B8GHP5_METPE]), DacW (*Euryarchaeota archaeon* [A0A1V5Z8G7_9EURY], *Methanospirillum hungatei* [Q2FM57_METHJ]) and DacX (*Methanoregula* *boonei* [A7I8S2_METB6], *Methanoregulaceae archaeon* [A0A1V4U3L9_9EURY], *Euryarchaeota archaeon* [A0A1V5ST82_9EURY] were included. From the other main branches, representatives of the bacterial orders Myxococcales (*Myxococcus virescens* [WP_090495195.1], *Stigmatella erecta *[WP_093522591.1], *Myxococcus stipitatus* (WP_015350295.1), *Stigmatella aurantiaca* [EAU67761.1]), Planctomycetales (*Planctomicrobium piriforme* [WP_092053393.1], *Isosphaera pallida* [WP_013566515.1], *Rhodopirellula islandica* [WP_047812869.1]) and Gemmatimonadales (*Gemmatimonadetes bacterium* [A0A2E4MZL2_9BACT]) were included. As members of the identified archaeal branches, organism from the following classes were included: Methanomicrobia (*Methanolobus psychrophilus* [WP_015053149.1], *Methanosarcina mazei* [AGF97235.1], *Methanocella conradii* [WP_014406877.1]), Archaeoglobi (*Ferroglobus placidus* [WP_012964775.1], *Archaeoglobus profundus* [WP_012940054.1]), the DHVE2 group (*Aciduliprofundum *sp. [WP_015283473.1]), Halobacteria (*H. volcanii* [WP_004041551.1], *Haloferax mediterranei* [WP_004056629.1], *Natrinema pallidum* [WP_006184372.1], *Halococcus thailandensis* [WP_007742370.1], *Halorubrum hochstenium* [WP_008580891.1], *Haloquadratum walsbyi* [WP_011571793.1], *Haloterrigena turkmenica* [WP_012943624.1], *Haloparvum sedimenti* [WP_058366734.1], *Haladaptatus cibarius* [WP_049970384.1], *Halogranum amylolyticum* [WP_089824883.1]), Thermococci (*Thermococcus kodakarensis* [WP_011249465.1], *Thermococcus paralvinellae* [WP_042679771.1], *Pyrococcus furiosus* [WP_011011999.1], *Pyrococcus horikoshii_OT3* [BAA30112.1], *Thermococcus litoralis* [WP_004066846.1]) and Methanococci (*Methanococcus voltae* [D7DR02_ METV3], *Methanocaldococcus vulcanius* [C9RHV3_METVM], *Methanococcus aeolicus* [A6UTS6_ META3], *Methanococcus maripaludis* [WP_011867877.1], *Methanocaldococcus jannaschii *[WP_010870515.1]). The adenylate cyclase domains of these sequences were again aligned with MUSCLE, and from this alignment a bootstrap consensus tree was inferred from 500 replicates (Felsenstein, 1985) and taken to represent the evolutionary history of the taxa analyzed (Felsenstein, 1985). Initial tree(s) for the heuristic search were obtained automatically by applying Neighbor‐Join and BioNJ algorithms to a matrix of pairwise distances estimated using a JTT model, and then selecting the topology with superior log likelihood value

Putative DAC genes are present in various euryarchaeal genomes and, similarly to bacterial DACs, they have been previously classified according to their attached domains (Corrigan & Gründling, 2013). Analysis of the genome of the euryarchaeal model organism *H. volcanii* DS2 (Hartman et al., 2010) revealed the presence of a single putative DAC encoded by the gene *dacZ* (HVO_1660) in an operon with *mscS2* (HVO_1659), a mechanosensitive channel of small conductance. An alignment of DacZ from *H. volcanii* and several putative DacZs from euryarchaeal model organisms with the biochemically and structurally characterized bacterial DAC CdaA from *L. monocytogenes* (Rosenberg et al., 2015) showed strong conservation of the DAC domain (Figure 1) with especially conservation of the catalytically important residues, like the highly conserved DGA motif followed by three hydrophobic residues (Rosenberg et al., 2015) (Figure 1, orange rectangles). No homology between DacZ from *H. volcanii* and CdaA from *L. monocytogenes* is found in the *N*‐terminal region. DACs of the DacA/CdaA family contain transmembrane domains at their *N*‐terminus (Corrigan & Gründling, 2013; Rosenberg et al., 2015), while until now no typical *N*‐terminal domain was identified for DacZ from *H. volcanii* which grouped this protein with other archaeal DacZ proteins (Corrigan & Gründling, 2013). To analyze the relation of DacZ from *H. volcanii* with other DACs, sequences related to DacZ and representatives from previously identified bacterial and archaeal classes were collected, the adenylate cyclase domains were aligned, and a phylogenetic tree was constructed (Figure A1). This analysis revealed that the adenylate cyclase domains of proteins of the DacZ and DacY/CdaZ classes are found in a similar branch of the tree, which is distinct from other bacterial and archaeal classes, and that this branch also contained several bacterial proteins. Homologs were identified in most euryarchaeal species, but no DACs were identified in crenarchaeota. Proteins of the DacY/CdaZ class, like CdaZ from *M. jannaschii*, contain an *N*‐terminal pyruvate kinase C‐terminal alpha/beta domain (PK_C) (Commichau et al., 2018; Corrigan & Gründling, 2013). Remarkably, although the conservation of the *N*‐terminal primary sequence of DacZ proteins was very low, homology modeling of the 3D structure of the *N*‐termini of these proteins indicated that members of the DacZ class and of the identified bacterial close homologs have a predicted fold similar to the *N*‐terminus of members of the DacY/CdaZ class. Thus, although they have low sequence similarity, DACs of the DacY/CdaZ and DacZ class most likely belong to a single widespread family of DACs sharing the common feature of an *N*‐terminal domain with a fold similar to the C‐terminal alpha/beta domain of pyruvate kinase.

### 3.2 c‐di‐AMP is present in H. volcanii grown in different media and at different growth phases

**Figure A2:**
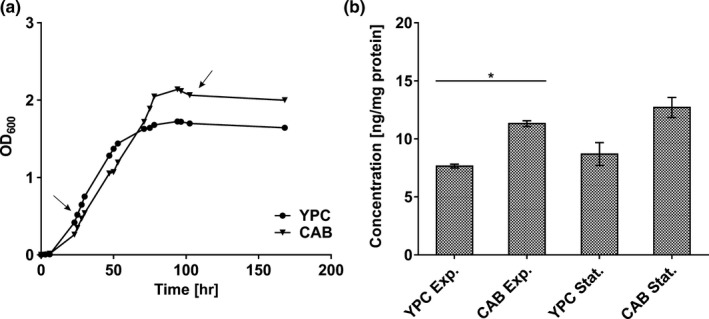
Measurement of cyclic di‐adenylate monophosphate (c‐di‐AMP) levels in *Haloferax volcanii* wild type grown under normal conditions in rich YPC and selective CAB medium. (a) Growth curves of wild type in YPC and CAB medium. Figure represents one of three biological replicates with similar results. Curves represent mean of three technical replicates. Arrows indicate time points of cell material sampling for cyclic nucleotide extraction. (b) Levels of c‐di‐AMP detected for *H. volcanii* wild type normalized to the protein content of each sample. Column values represent average of two biological replicates with three technical replicates each. Error bars indicate standard deviation. Statistical analysis was performed using a two‐tailed Student's *t* test with a critical p‐value for significance corrected according to Bonferroni (*p*‐value: 0.05/6 [number of statistical tests] = 0.0083). Asterisk (*) indicates a significant difference (*p*‐value < 0.0083) (YPC Exp. vs. CAB Exp.: 0.0034). (Exp.: exponential; Stat.: stationary)

The presence of a putative DAC in the genome of *H. volcanii* suggested that c‐di‐AMP might be present in this species as well. To answer this question, *H. volcanii* wild type strain (H26) (Allers et al., 2004) was grown to exponential and stationary phase in rich YPC and selective CAB media (Figure A2a), and cell pellet extracts were analyzed for the presence of c‐di‐AMP. For both media and growth phases, comparable amounts of c‐di‐AMP were detected (Figure A2b), however, with a tendency for increased c‐di‐AMP concentrations in cells grown in CAB medium. Combining two biological replicates (with three technical replicates each), a mean c‐di‐AMP concentration for growth in YPC of 8.17 ± 0.73 ng/mg protein was measured. For growth in CAB medium, a value of 12.01 ± 0.31 ng/mg protein was determined.

### 3.3 *Heterologously expressed DacZ shows manganese‐dependent *in vitro* DAC activity*

**Figure 2:**
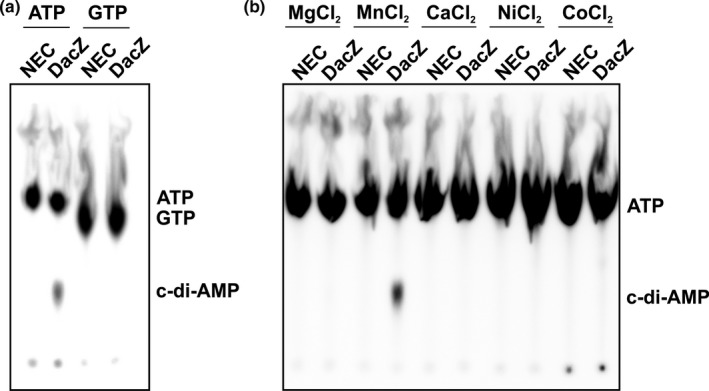
DAC activity measurements for substrate specify and metal cofactor usage of DacZ purified from *Escherichia coli*. (a) Phosphor screen of thin‐layer chromatography separating enzymatic products of in vitro DAC activity of DacZ using [α‐^32^P]ATP, respectively, [α‐^32^P]GTP as substrate. Figure represents one of three technical replicates with similar results. (b) Phosphor screen of thin‐layer chromatography separating enzymatic products of in vitro DAC activity of DacZ incubated with different metal‐(II) chloride solutions using [α‐^32^P]ATP as substrate. Figure represents one of three technical replicates with similar results. Position of cyclic di‐adenylate monophosphate (c‐di‐AMP) is indicated based on previous results from comparable assays (Bai et al., 2012) (NEC: non enzyme control)

**Figure A3:**
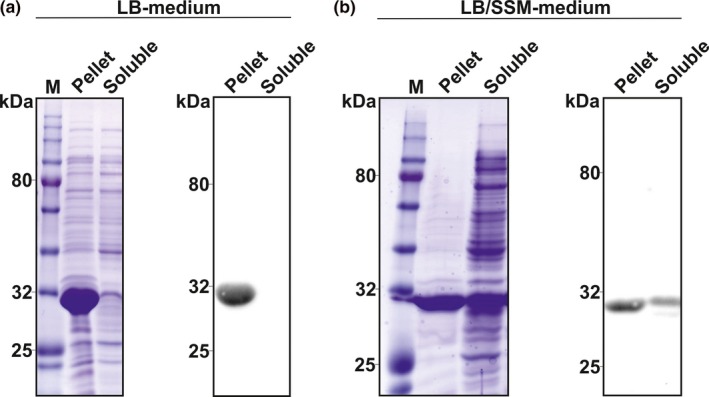
Analysis of heterologous expressed DacZ in *Escherichia coli* grown in LB‐ or LB/SMM‐medium. (a) SDSPAGE (left) and α‐histidine Western Blot (right) of pellet (insoluble) and soluble fraction of lysed *E. coli* cells expressing 10x histidine‐tagged DacZ (molecular weight 10x His‐DacZ: 30.3 kDa) grown in standard LB medium (M: molecular weight marker). (B) SDS‐PAGE (left) and α‐histidine Western Blot (right) of pellet (insoluble), and soluble fraction of lysed *E. coli* cells expressing 10x histidine‐tagged DacZ grown in osmotically active LB/SMM‐medium

**Figure A4:**
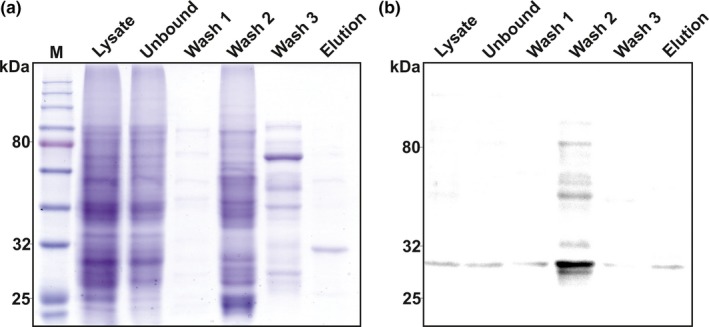
Heterologous expression and purification of *Haloferax volcanii* DacZ in *Escherichia coli*. (a) SDS‐PAGE of lysate, unbound, wash 1‐3, and elution fractions following purification of 10x histidine‐tagged DacZ (molecular weight 10x His‐DacZ: 30.3 kDa) (M: molecular weight marker). (b) α‐histidine Western blot of lysate, unbound, wash 1‐3, and elution fractions

In order to investigate whether the *H. volcanii dacZ* encodes an active DAC, the coding sequence of *dacZ* was cloned into the expression vector p7XNH3, which adds a 10x histidine‐tag to the *N*‐terminus of the protein. Expression in conventional LB medium did not yield any soluble protein (Figure A3a) as the high‐salt environment adapted DacZ from *H. volcanii* most likely misfolded in the cytoplasm of *E. coli*. However, it is known that the addition of osmotically active substances (i.e*.,* sucrose) to the medium increases the intracellular concentration of K^+^ ions and organic counterions in *E. coli* (Lucht & Bremer, 1994; McLaggan, Naprstek, Buurman, & Epstein, 1994). This increased intracellular osmolarity is likely to assist the proper folding of proteins from halophilic organisms as their native hosts naturally exhibit increased osmolarities in their cytoplasm as well (Oren, 2008). Indeed, the expression in osmotically active LB/SMM medium (see Section 2) led to soluble expression of DacZ in *E. coli* (Figure A3b). DacZ was purified by Ni‐affinity chromatography (Figure A4a,b) and assayed for in vitro DAC activity. Using α‐^32^P‐labeled ATP as a substrate, the synthesis of c‐di‐AMP (DAC activity) was observed for purified DacZ (Figure 2a). This DAC activity was specific for ATP as substrate (Figure 2a) and manganese (II)‐ions as cofactor (Figure 2b). In addition to TLC with radioactive [α‐^32^P]ATP, the formation of c‐di‐AMP by DacZ was also confirmed by mass spectrometry. 10 µmol/L DacZ obtained from three independent purifications was incubated with ATP for 1 hr at 37°C, which resulted in the production of a mean c‐di‐AMP concentration of 6.01 ± 0.11 µmol/L (with three technical replicates for each enzyme batch). Notably, *E. coli*, which was used as heterologous host for protein expression, does not possess any DACs (Corrigan et al., 2011). Therefore, the DAC activity observed in vitro originated from the haloarchaeal DacZ, confirming its identity as c‐di‐AMP producing DAC.

### 3.4 c‐di‐AMP is essential and its levels need to be tightly regulated

**Figure A5:**
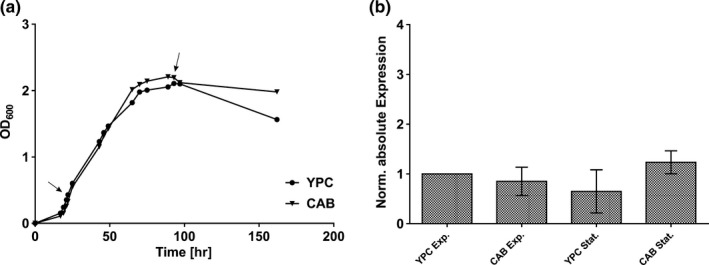
Comparison of dacZ transcription in *Haloferax volcanii* wild type grown under normal conditions in rich YPC and selective CAB medium. (a) Growth curves of wild type in YPC and CAB medium. Figure represents one of three biological replicates with similar results. Curves represent mean of three technical replicates. Arrows indicate time points of cell material sampling for RNA extraction. (b) Normalized absolute expression levels of dacZ in wild type grown in YPC and CAB medium. Column values represent average of three biological replicates with three technical replicates each. Error bars indicate standard deviation. Absolute expression was calculated using a standard curve of Cq‐values obtained for a defined amount of dacZ target sequence. Absolute expression levels for conditions YPC stationary growth and CAB exponential and stationary growth were normalized to expression in YPC exponential cells (set to 1). (Exp.: exponential; Stat.: stationary)

**Figure A6:**
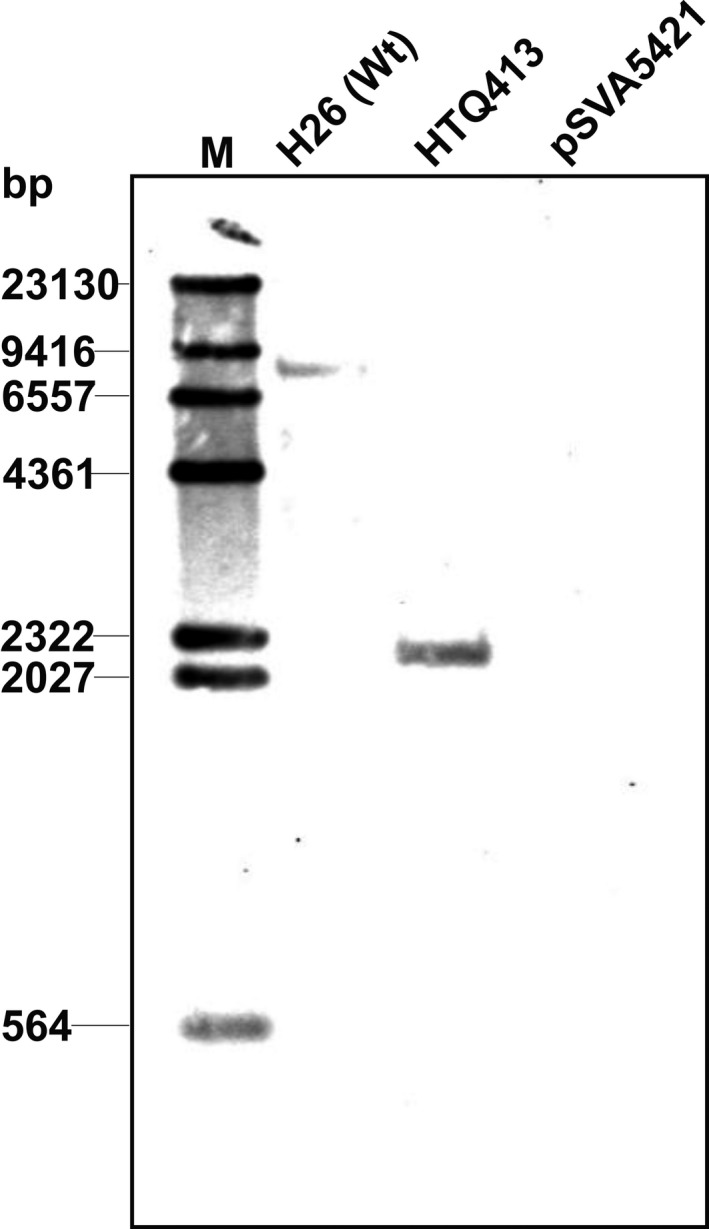
Southern blot detection of complete replacement of dacZ from its chromosomal locus by trpA gene (=Strain HTQ413) using a DIG‐labeled DNA probe against a flanking region within HVO_1659 that was not present on the *trp* ^+^‐marked deletion construct pSVA5421. The genomic DNA of wild type and genomic dacZ deletion strain was digested with EcoRV and StuI. Expected size of target fragments differs due to a unique StuI site in the trpA gene: Wild type: 7,825 bp; HTQ413: 2,128 bp. The *trp* ^+^‐marked deletion construct pSVA5421 was included as control. (M: molecular weight marker)

**Figure A7:**
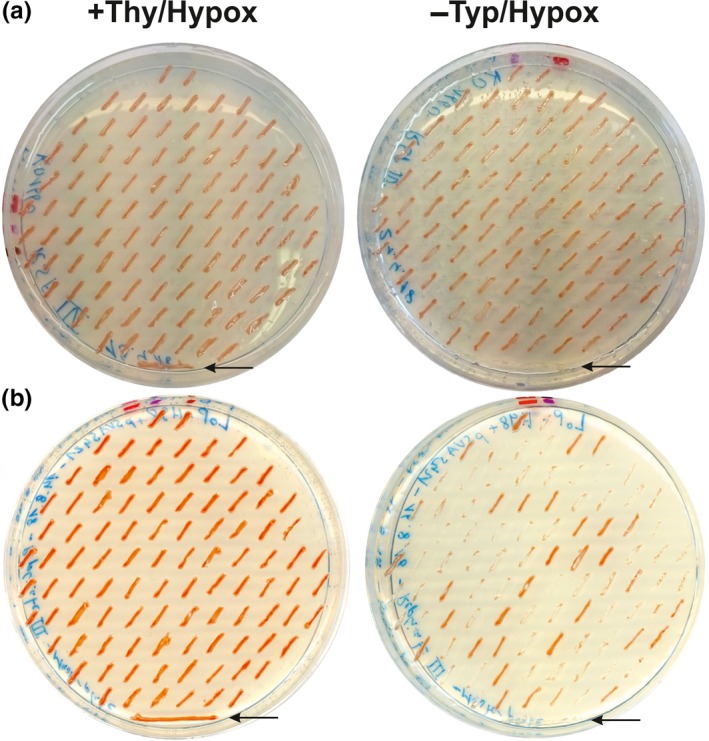
Loss of pSVA5421 (p[dacZ+hdrB+]) from HTQ413 (genomic dacZ deletion strain) and H98 (ΔpyrE2; ΔhdrB) (control) after unselective growth in CAB medium with thymidine & hypoxanthine. Figure represents one of three biological replicates with similar results. (a) Unselectively grown HTQ413 colonies (left) and growth of identical colonies on selective plate (right). Position of a colony within the grid pattern on the left plate is identical to the colonies' position on the right plate. Arrow indicates position of untransformed H98 as control strain. (b) Unselectively grown pSVA5421‐transformed H98 colonies (left) and growth of identical colonies on selective plate (right). Arrows indicate position of untransformed H98 as control strain

Several studies showed that DAC genes in bacteria are essential and cannot be deleted under standard growth conditions (Commichau & Stülke, 2018; Corrigan & Gründling, 2013; Fahmi, Port, & Cho, 2017). Having validated the identity of *dacZ* as gene encoding a DAC in *H. volcanii* the question arose whether this gene is also essential for this archaeal species. Using qPCR, the expression profile of *dacZ *was analyzed in identical growth conditions as used for the extraction and isolation of c‐di‐AMP. The normalized absolute expression levels of *dacZ* were similar for exponential and stationary growth in rich YPC and selective CAB media (Figure A5), suggesting a constitutive expression of the gene under these standard conditions. The construction of a single gene deletion mutant of *dacZ* was then attempted. Comparable to results in various bacteria, it was not possible to obtain a Δ*dacZ* strain via the standard pop‐in/pop‐out method (Allers et al., 2004). In all halobacteria, the *dacZ* gene is found in an operon with the *mscS2* gene (Figure A1). Deletion of the predicted *dacZ* + *mscS2* operon also failed, but a single gene deletion of *mscS2* could be obtained (Strain: HTQ412). These findings indicate that *dacZ* is an essential gene. To prove its essentiality, the *dacZ *gene was deleted from its chromosomal locus in a triple auxotrophic background strain (Δ*pyrE2* Δ*trpA* Δ*hdrB*; Strain: H99) using a *trp* ^+^‐marked deletion construct, in the presence of *dacZ* on a low‐copy replicative plasmid marked with *hdrB* ([p‐*dacZ* ^+^ *hdrB* ^+^]) to complement the genomic deletion *in trans*. Successful deletion of *dacZ* from its chromosomal locus was confirmed by restriction digest (using a unique *Stu*I site within the *trpA* gene that replaced *dacZ* at its native locus) and subsequent Southern Blot analysis (Figure A6). The resulting chromosomal *dacZ* deletion strain (∆*pyrE2* ∆*trpA* ∆*hdrB* ∆*dacZ*::*trpA* ^+^ p[*dacZ* ^+^ *hdrB* ^+^]; Strain: HTQ413) was grown in medium containing thymidine and hypoxanthine, which relieves selective pressure for *hdrB*. When nonselectively cultivated cells of the chromosomal *dacZ* deletion strain were transferred to selective plates without thymidine and hypoxanthine, no auxotrophic colonies were obtained (Figure A7a), indicating that the complementing plasmid [p‐*dacZ* ^+^ *hdrB* ^+^] could not be lost. As corresponding control experiment, a double auxotrophic background strain (Δ*pyrE2* Δ*hdrB*; Strain: H98) was also transformed with the *dacZ*‐complementing plasmid [p‐*dacZ* ^+^ *hdrB* ^+^] and subsequently subjected to identical unselective growth conditions. In these control experiments, several colonies auxotrophic for thymidine and hypoxanthine could be detected (Figure A7b). In three biological replicates of the control experiments, a mean loss‐of‐plasmid‐frequency of 64.56 ± 2.05% was determined. This proved that retention of the plasmid‐encoded *dacZ* gene is essential for the genomic *dacZ* deletion strain (Lestini et al., 2010).

**Figure 3:**
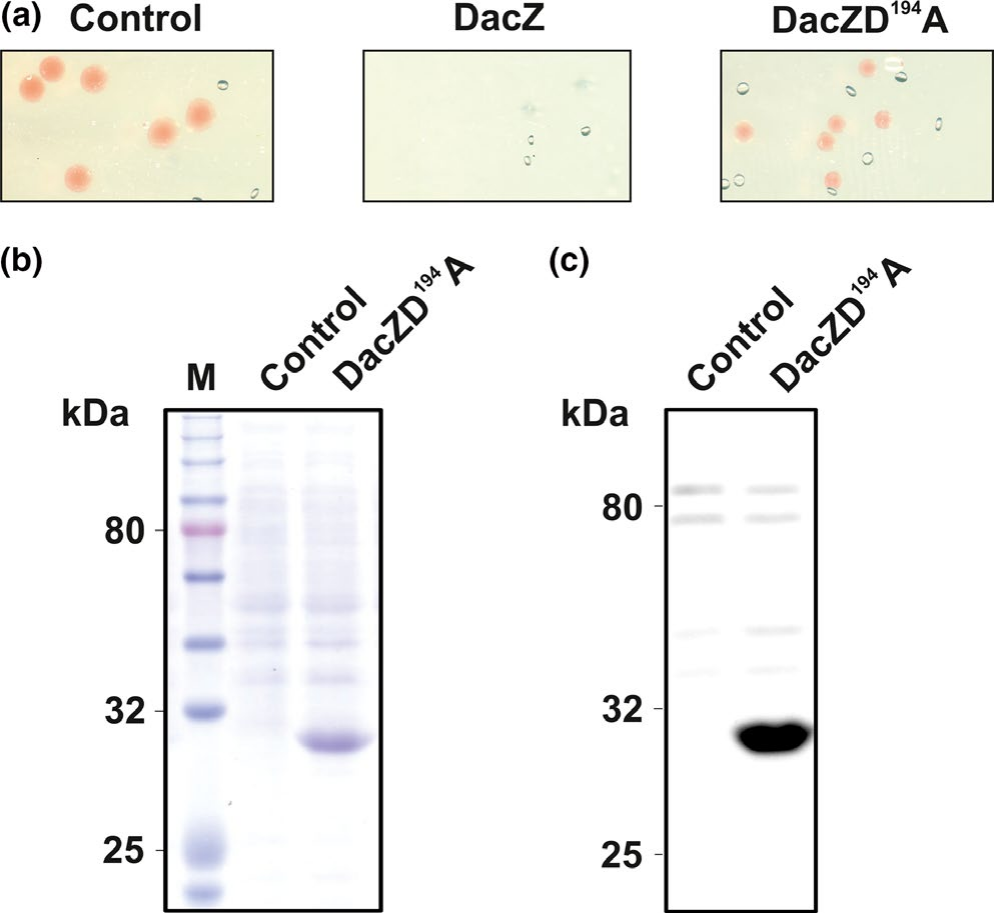
Homologous overexpression of DacZ and DacZD^194^A in *Haloferax volcanii* H1424. Figure represents one of three biological replicates with similar results. (a) Plate sections of H1424 transformed with empty backbone control (pTA1991), 6x histidine‐tagged DacZ overexpression construct (pSVA5417) and 6x histidine‐tagged DacZD^194^A overexpression construct (pSVA5433). (b) SDS‐PAGE of total cell pellet of H1424 cells transformed with empty backbone control or DacZD^194^A construct (Molecular weight 6x His‐DacZ: 29.8 kDa). (c) α‐histidine Western blot of total cell pellet of H1424 cells transformed with empty backbone control or DacZD^194^A construct. Bands appearing in both lines are unspecific bands characteristic for *H. volcanii*. (M: molecular weight marker)

**Figure A8:**
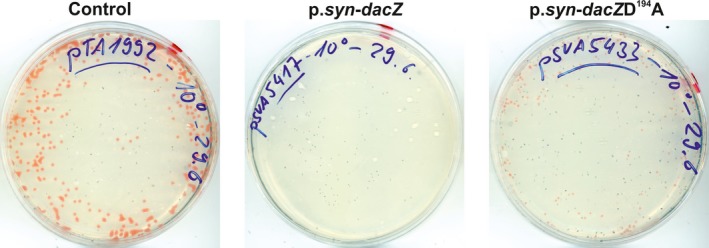
Complete plates of H1424 transformed with the empty backbone control (pTA1992; p.syn‐), *DacZ* overexpression construct (*pSVA5417*; p.*syn‐dacZ*) and *DacZD_194_A* overexpression construct (*pSVA5433*; p.*syn‐dacZD194A*). Plates contain sections presented in main figure 4 and represent a biological replicate of three independent experiments with similar results

The fact that the *dacZ* gene is essential indicates that a certain level of c‐di‐AMP is essential for the viability of *H. volcanii*. For bacteria, it has been reported that increased levels of c‐di‐AMP have a harmful effect on the cells (Gundlach et al., 2015). To determine if this is also the case in *H. volcanii*, a plasmid for homologous overexpression of DacZ was constructed where the *dacZ* gene is under the control of a strong constitutively active promotor (p.*syn*) (Large et al., 2007). Transformation of a *H. volcanii* strain (H1424) that has been optimized for protein expression (Stroud, Liddell, & Allers, 2012) with this construct did not yield any transformed cells (Figure 3a; Figure A8), whereas an empty vector control gave several transformants (Figure 3a; Figure A8). A single amino acid mutation (D^194^→A) in the highly conserved D^194^GA motive of DacZ (Figure 1) was introduced in the overexpression plasmid as such a mutation is expected to abolish DAC activity (Rosenberg et al., 2015). This construct could be transformed into *H. volcanii* and yielded positive colonies, but they were smaller than those containing the empty vector control (Figure 3a; Figure A8). The expression of DacZ(D^194^A) was also confirmed by SDS‐PAGE and Western Blot analysis, making use of a 6x histidine‐tag fused to the *N*‐terminus (Figure 3b,c). This result suggested that increased expression of wild type DacZ leading to increased intracellular c‐di‐AMP levels is lethal for *H. volcanii*.

### 3.5 Changed intracellular c‐di‐AMP levels causes increased cell volume in medium with low sodium chloride concentration

**Figure 4:**
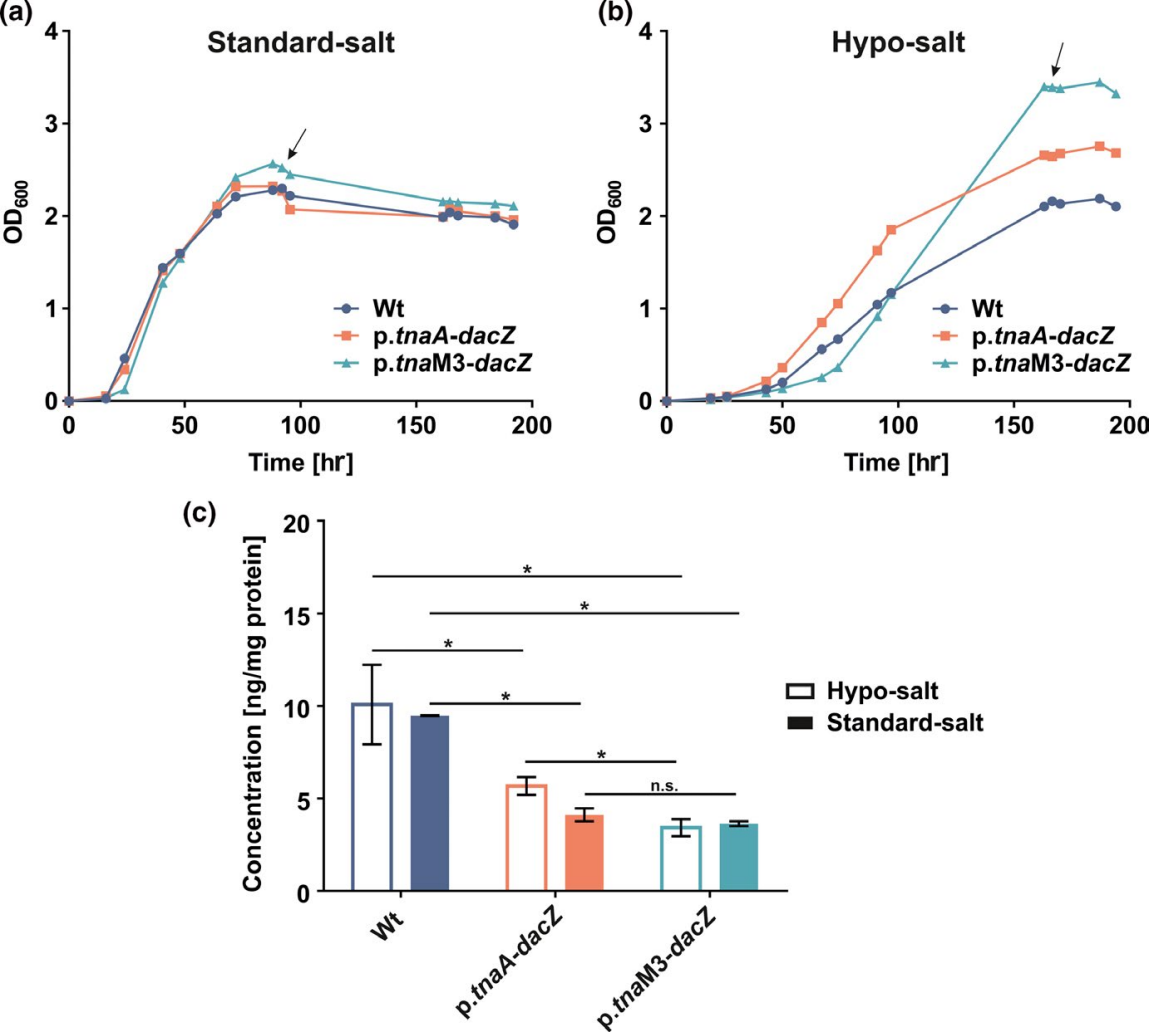
Measurement of cyclic di‐adenylate monophosphate (c‐di‐AMP) levels in *Haloferax volcanii* wild type (Wt), p.*tnaA*‐*dacZ* strain and p.*tna*M3‐*dacZ* strain grown to stationary phase in hypo‐ (1.8 M NaCl) and standard‐salt (2.5 M NaCl) CAB medium. (a) Growth curves of wild type, p.*tnaA*‐*dacZ* strain and p.*tnaM3‐dacZ* strain in standard‐salt CAB medium. Figure represents one of five biological replicates with similar results. Curves represent mean of three technical replicates. Arrow indicates time point of cell material sampling for cyclic nucleotide extraction. (b) Growth curves of wild type, p.*tnaA*‐*dacZ* strain and p.*tna*M3‐*dacZ* strain in hypo‐salt CAB medium. Figure represents one of five biological replicates with similar results. Curves represent mean of three technical replicates. Arrow indicates time point of cell material sampling for cyclic nucleotide extraction. (c) Levels of c‐di‐AMP detected for wild type, p.*tnaA*‐*dacZ* strain and p.*tna*M3‐*dacZ* strain normalized to the protein content of each sample. Column values represent average of four biological replicates (hypo‐salt conditions), respectively, two biological replicates (standard‐salt conditions) with three technical replicates each. Error bars indicate standard deviation. Statistical analysis was performed using a two‐tailed Student's *t* test with a critical *p*‐value for significance corrected according to Bonferroni (*p*‐value: 0.05/3 (number of statistical tests) = 0.016). Asterisk (*) indicates a significant difference (*p*‐value < 0.016) (Hypo‐salt: Wt vs. p.*tnaA*‐*dacZ*: 0.0071; Wt vs. p.*tna*M3‐*dacZ*: 0.0009; p.*tnaA*‐*dacZ* vs. p.*tna*M3‐*dacZ*: 0.0005; Standard‐salt: Wt vs. p.*tnaA*‐*dacZ*: 0.0021; Wt vs. p.*tna*M3‐*dacZ*: 0.0003). n.s.: not significant. For reasons of clarity statistical analysis for the comparison of a strain in hypo‐ and standard‐salt medium is excluded from the figure. Using a two‐tailed Student's *t* test with a critical *p*‐value for significance of 0.05 only the p.*tnaA*‐*dacZ *strain showed a significant (*p*‐value: 0.016) difference in these two different media)

Because neither gene deletion nor overexpression of *dacZ* had been possible, a promotor exchange approach (Skowyra & MacNeill, 2012) was used to examine the effects of altered intracellular c‐di‐AMP levels. Two mutant strains were created where the native promotor of *dacZ* was replaced with the tryptophan‐inducible promotor (p.*tnaA*) of the tryptophanase gene *tnaA* (HVO_0789) (Large et al., 2007) or, respectively, with a genetically engineered version of p.*tnaA* with ~50% reduced activity (with regard to p.*tnaA*) (p.*tna*M3) (MacNeill, unpublished data). The expression levels of *dacZ* were altered to the basal activity of p.*tnaA* (Strain: HTQ406) and p.*tna*M3 (Strain: HTQ410), respectively. These two mutant strains showed no growth phenotype in medium with the standard concentration of NaCl (2.5 M) (Figure 4a). To investigate how the respective exchange of the native *dacZ* promotor influences the c‐di‐AMP concentration in the p.*tnaA*‐ and p.*tna*M3‐induced *dac*Z‐expressing strains, cell extracts from standard‐salt medium stationary grown cultures were analyzed for their c‐di‐AMP content (Figure 4c). A comparison of the strains with the wild type revealed that, even though there was no obvious growth phenotype visible, they exhibit significantly reduced c‐di‐AMP levels. Especially the p.*tna*M3‐induced strain showed considerably lower intracellular c‐di‐AMP levels. This observation suggested that due to the replacement of the promotor of *dacZ* with p.*tnaA* and p.*tna*M3, respectively, the expression of DacZ was reduced which in turn led to decreased c‐di‐AMP concentrations within the two mutant strains.

**Figure 5:**
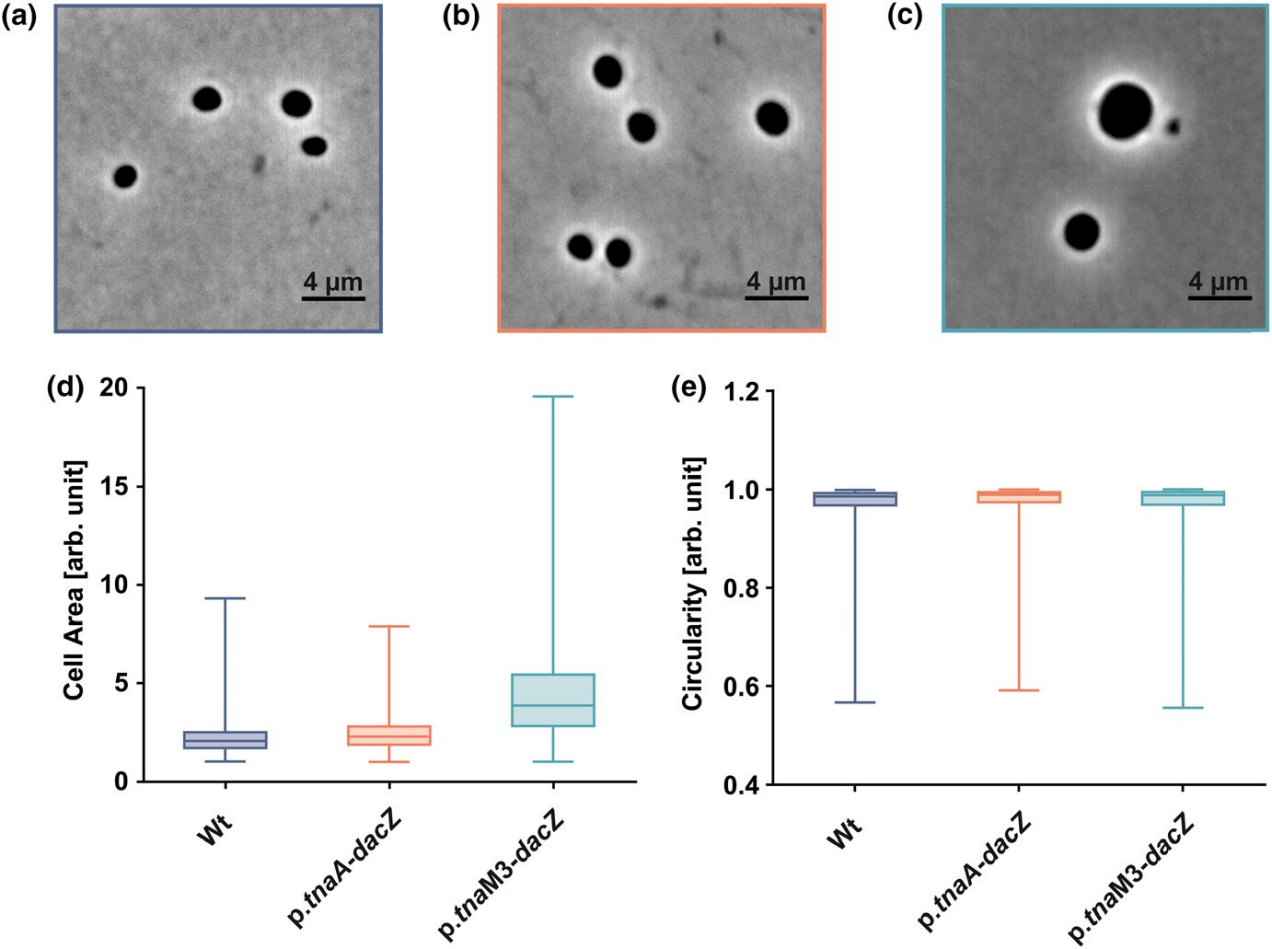
Phase‐contrast light microscopy and cell shape evaluation of *Haloferax volcanii* wild type, p.*tnaA*‐*dacZ* strain and p.*tna*M3‐*dacZ* strain grown to stationary phase in hypo‐salt CAB medium (1.8 M NaCl). (a) Representative phase‐contrast image of wild type cells at a magnification of 1000x. Scale bar of 4 µm is indicated in the picture. (b) Representative phase‐contrast image of p.*tnaA*‐*dacZ* cells at a magnification of 1000x. (c) Representative phase‐contrast image of p.*tna*M3‐*dacZ* cells at a magnification of 1000x. (d) Cell area distribution for wild type, p.*tnaA*‐*dacZ* strain and p.*tna*M3‐*dacZ* strain. Boxes represent values from three biological replicates with >500 cells analyzed for each strain. Whiskers indicate the full range of variation (from minimal to maximal value). Band inside the box indicates the median. Higher values indicate increased cell area. (e) Cell circularity distribution for wild type, p.*tnaA*‐*dacZ* strain and p.*tna*M3‐*dacZ* strain. Values close to one indicate that cells resemble a circle. Boxes represent values from three biological replicates with >500 cells analyzed for each strain. Whiskers indicate the full range of variation (from minimal to maxima value). Band inside the box indicates the median

**Figure A9:**
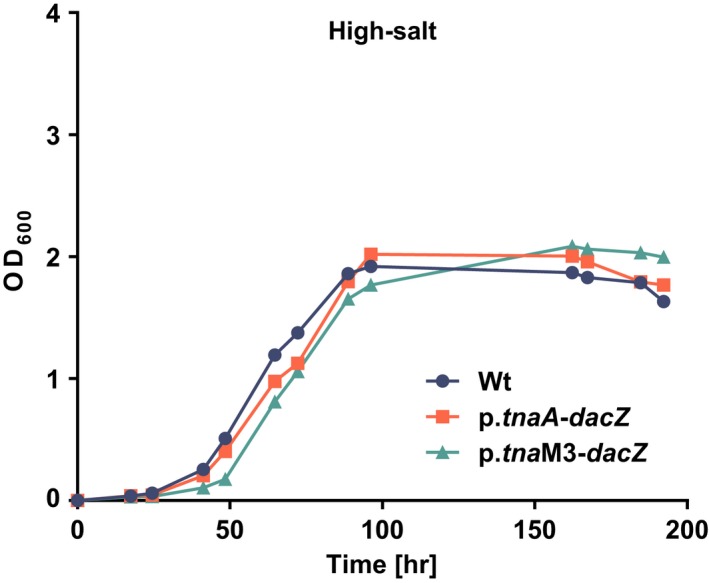
Growth of H. volcanii wild type, p.*tnaA‐dacZ* strain and p.*tnaM3‐dacZ* strain in high‐salt (4.5 M NaCl) medium. Figure represents one of five biological replicates with similar results. Curves represent mean of three technical replicates

The conservation of a predicted operon containing a DAC and an *msc* gene in various Haloarchaea suggests that c‐di‐AMP‐related signaling might be involved in the regulation of osmotic homeostasis. In order to test this hypothesis, the p.*tnaA*‐*dacZ* strain, the p.*tna*M3‐*dacZ* strain, and the wild type as a control were subjected to growth in CAB medium with low (1.8 M: hypo‐salt) and increased (4.5 M: high‐salt) NaCl concentration. All three strains exhibited equal growth in high‐salt medium (Figure A9). However, in hypo‐salt medium, a growth phenotype for both mutants was observed (Figure 4b). In comparison to the wild type, the p.*tnaA*‐*dacZ* strain exhibited in general slightly elevated OD_600_ values in this condition. The p.*tna*M3‐induced mutant had a different phenotype as it exhibited a prolonged lag phase followed by an extended exponential phase that peaked at an increased maximal OD. Additional analysis of the c‐di‐AMP levels of hypo‐salt medium stationary grown wild type, p.*tnaA*‐*dacZ*, and p.*tna*M3‐*dacZ* cultures closely resembled the levels measured for growth in standard‐salt medium (Figure 4c). All three strains exhibited similar concentrations of c‐di‐AMP for growth in hypo‐ and standard‐salt medium with a slight tendency for increased c‐di‐AMP levels in hypo‐salt conditions. However, only for the p.*tnaA*‐induced strain, a significant difference between hypo‐ and standard‐salt c‐di‐AMP levels could be detected. The observation that the decreased intracellular c‐di‐AMP levels of the two mutant strains also occurred in hypo‐salt conditions suggested that the observed growth phenotypes are linked to the changes in the c‐di‐AMP levels. To investigate the hypo‐salt phenotype further, stationary phase cells were analyzed using phase‐contrast light microscopy. The cells of the p.*tna*M3‐induced mutant were round but had an increased cell area (Figure 5c), compared to the wild type (Figure 5a) and p.*tnaA*‐*dacZ* cells (Figure 5b). Quantification of cell area (Figure 5d) and circularity (Figure 5e) confirmed that p.*tna*M3‐*dacZ* cells were round (and not rod‐shaped and/or elongated which would also cause an increased cell area), like the wild type and p.*tnaA*‐*dacZ* cells, but in general bigger. Notably, there was a population among the analyzed p.*tna*M3‐induced cells that exhibited a greatly increased cell area, suggesting that the increased OD_600_ values observed for this strain were caused by a swelling of the cells rather than elevated growth levels.

**Figure A10:**
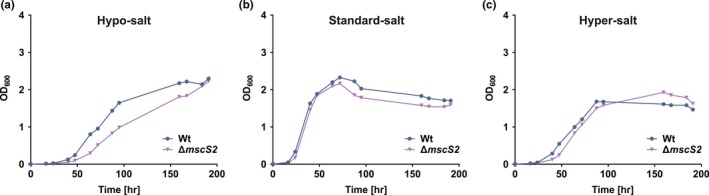
Growth of *Haloferax volcanii* wild type and ΔmscS2 in hypo‐, standard‐, and high‐salt medium. Figure represents one of three biological replicates with similar results. Curves represent mean of three technical replicates. (a) Growth curves of wild type and ΔmscS2 in CAB medium with 1.8 M NaCl (hypo‐salt). (b) Growth curves of wild type and ΔmscS2 in CAB medium with 2.5 M NaCl (standard‐salt). (c) Growth curves of wild type and ΔmscS2 in CAB medium with 4.5 M NaCl (high‐salt)

**Figure A11:**
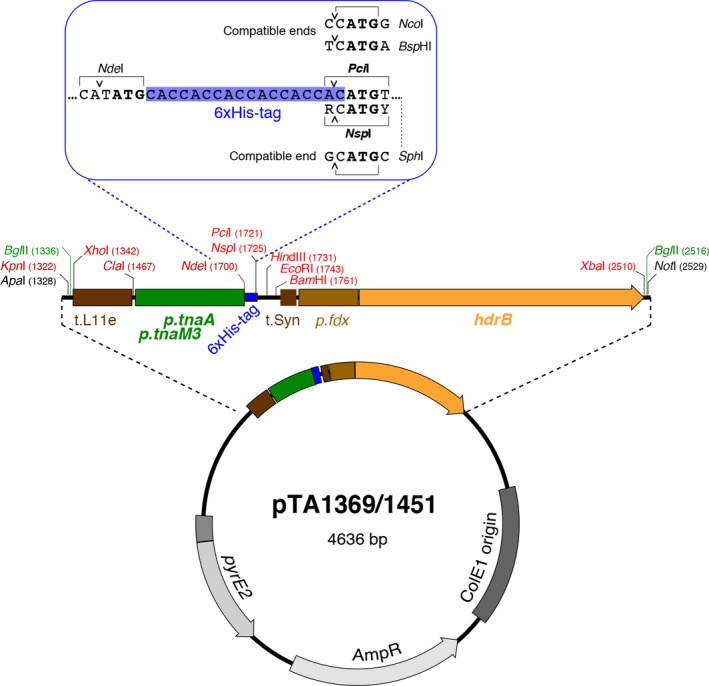
pTA1369 and pTA1451 are pyrE2‐marked vectors used to generate tryptophan‐inducible alleles of genes at their native chromosomal locus. They feature a multiple cloning site comprising a tryptophanase promotor p.tnaA in pTA1369 or a reduced activity p.tnaM3 promoter in pTA1451, an optional 6x His tag, and an hdrB marker under a ferredoxin promotor; the multiple cloning site is flanked by BglII sites, so that it can be inserted at the compatible BamHI site between the upstream and downstream flanking sequences of a gene deletion construct

As mentioned earlier, the conservation of a predicted operon of a DAC and an *msc* gene in haloarchaea suggests the involvement of c‐di‐AMP‐related signaling in osmotic homeostasis. To examine the role of the operonic *msc* gene and to exclude the possibility that the observed phenotypes of the p.*tnaA*‐*dacZ* strain and the p.*tna*M3‐*dacZ* strain were rather caused by changes in expression of *mscS2* than *dacZ*, the Δ*mscS2* strain was also subjected to growth in hypo‐, standard‐, and high‐salt medium. This strain showed no growth phenotype in standard‐ and high‐salt medium (Figure A10b,c). However, at hypo‐salt conditions, the strain showed a phenotype that was distinct from the phenotypes of the p.*tnaA*‐, respectively, p.*tna*M3‐induced strain as it exhibited slightly reduced growth (Figure A10a), suggesting an in general decreased adaption to low salt conditions.

## 4 DISCUSSION

This study provides the first direct evidence for the in vivo presence of c‐di‐AMP in an archaeal species. The haloarchaeal protein DacZ was identified as DAC and the corresponding gene *dacZ* was demonstrated to be essential, suggesting a vital function of c‐di‐AMP‐mediated signaling in *H. volcanii*. Growth experiments of *H. volcanii* strains with altered intracellular c‐di‐AMP levels showed distinct phenotypes and cell morphologies in hypo‐salt medium, but not in media with standard or increased NaCl concentration.

Various studies on intracellular c‐di‐AMP levels of bacteria have been conducted so far and a comparison with results presented here suggests that the intracellular concentration of c‐di‐AMP in *H. volcanii *(10.09 ng/mg protein on average under the tested conditions) is comparable to that reported for bacterial species. It was similar to levels in wild type *Bacillus subtilis *(5.6 ± 2.82 ng/mg protein) (Mehne et al., 2013), however, considerably lower than the measurements in *Staphylococcus aureus *(~140 ng/mg protein) (Bowman, Zeden, Schuster, Kaever, & Gründling, 2016).

All c‐di‐AMP‐producing DACs share a conserved DAC domain (also known as DisA_N) that catalyzes the reaction of two ATP to form c‐di‐AMP. Several different families of DACs have been identified, which all differ in their additional regulatory domain (Commichau et al., 2018; Corrigan & Gründling, 2013). So far, members of four bacterial DAC classes have been characterized in more detail: DisA (Witte et al., 2008), DacA/CdaA (Rosenberg et al., 2015), DacB/CdaS (Mehne et al., 2014), and CdaM (Blötz et al., 2017). Additionally, DAC activity has been demonstrated for a representative of the archaeal DacY/CdaZ class (Kellenberger et al., 2015). For bacterial DACs, it has been reported that the aspartic acid within the highly conserved DGA motif interacts with an ATP molecule bound to a second DAC molecule (Rosenberg et al., 2015). As this motif is also conserved in the haloarchaeal (and other euryarchaeal) DacZ it suggests that this DAC also forms dimers or higher oligomers to catalyze the formation of c‐di‐AMP. Contrary to biochemically characterized bacterial DAC representatives, which were reported to have a certain promiscuity for their metal cofactor (Bai et al., 2012; Rosenberg et al., 2015), DacZ from *H. volcanii* exhibited activity exclusively with manganese. A phylogenetic analysis of the DAC domain of proteins related to DacZ from *H. volcanii* showed that homologs are presents in almost all euryarchaea and in some bacteria and that, remarkably, structure prediction methods revealed that the *N*‐terminal domain has, despite low sequence homology, a fold that resembles the pyruvate kinase C‐terminal alpha/beta domain (PK_C) fold. This domain is also found in DAC representatives of the DacY/CdaZ class (Commichau et al., 2018; Corrigan & Gründling, 2013) suggesting that the archaeal DAC classes DacZ and DacY/CdaZ form one large family that is not exclusively found in archaea but also in some bacteria (Figure A1). The PK_C fold was initially identified to bind fructose‐1,6‐bisphosphate to allosterically regulate the activity of pyruvate kinase. Alternatively, the putative pyruvate kinase (AF0103) from *Archaeoglobus fulgidus* (PDB: 1VP8) was shown to bind flavin mononucleotide ([Link]). This suggests a regulatory role for this *N*‐terminal domain.

Various studies in bacterial model organisms have shown that both a complete deletion of c‐di‐AMP (by deletion of DACs) and increased levels of the nucleotide are lethal for cells under standard growth conditions, which has earned c‐di‐AMP the name of “an essential poison” (Gundlach et al., 2015). The results presented in this study suggest that this is also the case for *H. volcanii*. However, for species where c‐di‐AMP‐related signaling was described in more detail, conditions were found under which DAC(s) could be deleted (Gundlach et al., 2017; Whiteley et al., 2015). Therefore, it is possible that under certain conditions, a deletion of *dacZ*, and thereby c‐di‐AMP, in *H. volcanii* could be accomplished.

Nevertheless, for standard growth conditions c‐di‐AMP is essential, suggesting its involvement in a vital cellular function. In order to survive in their high salinity habitats, haloarchaea like *H. volcanii* concentrate high amounts of potassium chloride in their cytoplasm to balance the high osmolarity of the surrounding environment (Meury & Kohiyama, 1989; Thombre, Shinde, Oke, Dhar, & Shouche, 2016). Results from bacteria that demonstrated c‐di‐AMPs' involvement in osmotic regulation and the fact that a membrane channel lies within the conserved haloarchaeal DAC operon indicated that c‐di‐AMP might have an osmoregulatory function in *H. volcanii* as well. The results for growth of the p.*tna*M3‐*dacZ *strain with its reduced c‐di‐AMP levels in hypo‐salt medium support this assumption. Cells of this strain had an increased cell area (and thereby also volume) which suggests an increased influx of medium/liquid to the cytoplasm. This observation matches with current models in bacteria where mutant strains of various species with decreased c‐di‐AMP levels exhibited phenotypes which were thought to be caused by an uncontrolled influx of osmolytes (Commichau, Gibhardt, Halbedel, Gundlach, & Stülke, 2017). For the p.*tna*M3‐induced strain, this suggests an in general increased (in comparison to the wild type) import of osmotically active substances to the cytoplasm. Under standard growth conditions (and also in high‐salt medium), p.*tna*M3‐*dacZ* cells showed no phenotype despite their reduced c‐di‐AMP content (Figure 4a,c; Figure A9). For these conditions, an increased influx of osmolytes might not necessarily cause a swelling of the cells as the high osmolarity of the surrounding medium would balance the increased osmolarity of the cytoplasm. However, in hypo‐salt medium, an uncontrolled import of osmolytes would increase the osmotic imbalance between medium and cytoplasm leading to the proposed influx of medium/liquid to the cells. In addition, the p.*tnaA*‐induced strain, which exhibited also a trend to lower c‐di‐AMP levels than the wild type (Figure 4c), showed also a growth phenotype in hypo‐salt medium slightly resembling the one of p.*tna*M3‐*dacZ *cells. However, the phenotypical effects of the c‐di‐AMP level reduction in this strain were considerably weaker than what could have been expected given the fact that the p.*tnaA*‐induced strain exhibited only a slightly higher c‐di‐AMP content than p.*tna*M3‐induced cells. These results again suggest that c‐di‐AMP levels in *H. volcanii* are subjected to a tight regulation and that a slight deviation already causes perceptible effects. Comparing c‐di‐AMP levels of wild type cells grown in hypo‐ and standard‐salt medium, a tendency to an increase of intracellular c‐di‐AMP in hypo‐salt conditions could be observed. Even though the difference was statistically not significant it supports the theory that a decrease in c‐di‐AMP concentration leads to an uncontrolled osmolyte influx as, in turn, increased c‐di‐AMP levels in the wild type grown in hypo‐salt conditions would cause a reduced import of osmolytes equivalent to an adaption to the reduced osmolarity of the surrounding environment.

As the *mscS2* gene lies in a predicted operon with *dacZ*, a change in the promotor for *dacZ* would most likely also influence expression of the mechanosensitive channel. However, the fact that *mscS2* could be deleted under standard growth conditions, indicates that this DAC operon included channel protein is, at least, not one of the main targets of c‐di‐AMP signaling under the tested conditions. This is also in line with bacterial studies that showed that mechanosensitive channels are rather controlled by means of their expression rate (Booth, 2014; Edwards et al., 2012), suggesting a function of the *mscS2* gene under conditions where increased c‐di‐AMP levels, and thereby increased expression of the operon *dacZ* and *mscS2*, are necessary.

Future experiments will elucidate the targets of c‐di‐AMP in *H. volcanii* and uncover the regulatory mechanism, which seems to be important for the osmotic homeostasis in this organism.

## CONFLICT OF INTERESTS

None declared.

## AUTHORS CONTRIBUTION

F.B. designed the experiments and analyzed data under supervision from C.v.d.D. and S.V.A. Most of the experiments were performed by F.B., except for several growth experiments (performed by L.T.) and enzyme assays using radioactive‐labeled nucleotides (performed by C.v.d.D.). The manuscript was written by F.B. with input from C.v.d.D., T.A., and S.V.A. V.K. supervised LC‐MS/MS measurements of c‐di‐AMP. T.Q. helped in supervising the project and S.V.A. conceived the original idea.

## ETHICS STATEMENT

None required.

## Data Availability

No sequence or proteomic data need to be accessible for this manuscript.

## APPENDIX 1

**Table A1:** Strains used in this study

Strain name	Background strain	Genotype	Source/reference
*Haloferax volcanii*			
H26	–	∆*pyrE2*	Allers et al., 2004
H98	–	∆*pyrE2* ∆*hdrB*	Allers et al., 2004
H99	–	∆*pyrE2* ∆*trpA* ∆*hdrB*	Allers et al., 2004
H1424	–	∆*pyrE2* ∆*hdrB* *Nph‐pitA* ∆*mrr cdc48d‐Ct*	Stroud et al., 2012
HTQ92	H26	∆*pyrE2 dacZ* ^+^::[*dacZ* *pyrE2* ^+^]	This study
HTQ405	H98	∆*pyrE2* ∆*hdrB* *dacZ* ^+^::[p.*tna*‐*dacZ* *hdrB* ^+ ^ *pyrE2* ^+^]	This study
HTQ406	H98	∆*pyrE2* ∆*hdrB* p.*tna*‐*dacZ*‐*hdrB* ^+^	This study
HTQ407	H98	∆*pyrE2* ∆*hdrB* *dacZ* ^+^::[p.*tna*M3‐*dacZ* *hdrB* ^+^ *pyrE2* ^+^]	This study
HTQ408	H26	∆*pyrE2* Operon *dacZ+mscS2* ^+^::[∆*dacZ+mscS2 pyrE2* ^+^]	This study
HTQ409	H99	∆*pyrE2 dacZ* ^+^::[∆*dacZ*::*trpA* ^+^ *pyrE2* ^+^]	This study
HTQ410	H98	∆*pyrE2* ∆*hdrB* p.*tna*M3‐*dacZ*‐*hdrB* ^+^	This study
HTQ411	H26	∆*pyrE2 mscS2* ^+^::[∆*mscS2 pyrE2* ^+^]	This study
HTQ412	H26	∆*pyrE2* ∆*mscS2*	This study
HTQ413	H99	∆*pyrE2* ∆*trpA* ∆*hdrB* ∆*dacZ*::*trpA* ^+^ p[*dacZ* ^+^ *hdrB* ^+^]	This study
*Escherichia coli*			
10‐beta competent cells “TOP10”	–	Δ*(ara‐leu) 7697 araD139 fhuA *Δ*lacX74 galK16 galE15 e14‐ *ϕ*80*d*lacZ*Δ*M15 recA1 relA1 endA1 nupG rpsL *(Str^R^)* rph spoT1 *Δ*(mrr‐hsdRMS‐mcrBC)*	New England Biolabs
*dam^−^*/*dcm^−^* competent	–	*ara‐14 leuB6 fhuA31 lacY1 tsx78 glnV44 galK2 galT22 mcrA dcm‐6 hisG4 rfbD1 R(zgb210::Tn10) *Tet^S^ * endA1 rspL136 *(Str^R^)* dam13::Tn9 *(Cam^R^)* xylA‐5 mtl‐1 thi‐1 mcrB1 hsdR2*	New England Biolabs
Rosetta™(DE3) competent cells	–	*F* ^−^ *ompT hsdS*_B_(r_B_^‐^ m_B_^‐^) *gal dcm* (DE3) pRARE (Cam^R^)	Novagen

**Table A2:** Plasmids used in this study

Plasmid number	Description	Primers used	Enzymes used	Source/reference
pTA131	*pyrE2* marked integrative vector for knock‐outs in* Haloferax volcanii *(Amp^r^)	–	–	Allers et al., 2004
pTA298	Vector containing *trpA* marker under a ferredoxin promoter between two *BamH*I sites (Amp^r^)	–	–	Lestini et al., 2010
pTA356	*hdrB* marked shuttle vector with pHV1 replication origin (Amp^r^)	–	–	Norais et al., 2007
pTA409	*pyrE2* and *hdrB* marked shuttle vector with pHV1 replication origin (Amp^r^)	–	–	Delmas et al., 2009
pTA1228	*pyrE2* and *hdrB* marked expression vector with pHV2 replication origin and tryptophanase promoter p.*tnaA*, for tryptophan‐inducible gene expression of *N*‐terminal 6x His‐tagged protein (Amp^r^)	–	–	Brendel et al., 2014
pTA1369	*pyrE2* marked vector containing a tryptophan promotor (p.*tnaA*)‐multiple cloning site‐construct with a *hdrB* marker under a ferredoxin promotor between two *Bgl*II sites; used to generate a tryptophan‐inducible allele of gene (Amp^r^)	–	–	This study; derived from pTA131 and pTA1228 (see Figure A11)
pTA1392	*pyrE2* and *hdrB* marked expression vector pTA1228 with insertion of Strep II tag, for tryptophan‐inducible gene expression of *N*‐terminal 6x His‐tagged and/or C‐terminal StrepII‐tagged protein (Amp^r^)	–	–	Gamble‐Milner, 2016
pTA1451	*pyrE2* marked vector containing a tryptophan promotor with reduced leak activity (p.*tna*M3)‐multiple cloning site‐construct with a *hdrB* marker under a ferredoxin promotor between two *Bgl*II sites; used to generate a tryptophan‐inducible allele of gene (Amp^r^)	–	*Cla*I; *Bcl*I; *Nde*I	This study; p.*tna*M3 promoter from pNPM‐*tna*M3‐HfxMCM (S. MacNeill, unpublished)(see Figure A11)
pTA1992	*pyrE2* and *hdrB* marked expression vector with pHV2 replication origin and synthetic promoter (p.*syn*), for constitutive gene expression of *N*‐terminal 6x His‐tagged and/or C‐terminal StrepII‐tagged protein (Amp^r^)	p.synF; p.synR2	*Bst*BI; *Nde*I	This study
p7XNH3	Expression vector for *E. coli* using T7‐promotor with *N*‐terminal 10x His‐Tag (FX‐Cloning system) (Kana^r^)	–	–	Geertsma & Dutzler, 2011
pSVA3930	pTA131‐based deletion construct for *dacZ* containing ~500 bp upstream and downstream flanking regions ligated via *BamH*I site (Amp^r^)	8001; 8002; 8003; 8004	*Kpn*I; *BamH*I; *Xba*I	This study
pSVA5408	p7XNH3‐based expression vector for heterologous expression of *dacZ* with *N*‐terminal 10x His‐Tag in *Escherichia coli *(Kana^r^)	7644; 7645	*Sap*I	This study
pSVA5411	pTA1369‐based vector containing a tryptophan‐inducible (p.*tnaA*) allele of *dacZ* with a *hdrB* marker under a ferredoxin promotor between two *Bgl*II sites (Amp^r^)	7654; 7662	*Nde*I; *EcoR*I	This study
pSVA5414	pSVA3930‐based integrative vector with tryptophan‐inducible (p.*tnaA*) allele of *dacZ* with a *hdrB* marker under a ferredoxin promotor inserted between upstream and downstream flanking regions at *BamH*I site (Amp^r^)	–	*Bgl*II; *BamH*I	This study
pSVA5417	pTA1992‐based expression vector for homologous expression of *dacZ* with *N*‐terminal 6x His‐Tag in *H. volcanii *(Amp^r^)	7669; 7633	*Pci*I; *Nco*I; *EcoR*I	This study
pSVA5418	pTA131‐based deletion construct for predicted Operon *dacZ+mscS2 *containing ~500 bp upstream and downstream flanking regions ligated via *BamH*I site (Amp^r^)	8001; 7672	*Kpn*I; *BamH*I; *Xba*I	This study
pSVA5419	pTA1451‐based vector containing a tryptophan‐inducible (p.*tna*M3) allele of *dacZ* with a *hdrB* marker under a ferredoxin promotor between two *Bgl*II sites (Amp^r^)	–	*Nde*I; *Xho*I	This study
pSVA5420	pSVA3930‐based integrative vector marked with *trpA* under a ferredoxin promoter ligated at *BamH*I site (Amp^r^)	–	*BamH*I	This study
pSVA5421	pTA356‐based episomal vector containing *dacZ *with ~1,000 bp upstream and downstream flanking regions (Amp^r^)	7680; 7681	*Kpn*I; *BamH*I	This study
pSVA5422	pTA409‐based episomal vector containing *dacZ *with ~1,000 bp upstream and downstream flanking regions (Amp^r^)	7680; 7681	*Kpn*I; *BamH*I	This study
pSVA5427	pTA131‐based deletion construct for *mscS2* containing ~500 bp upstream and downstream flanking regions ligated via *BamH*I site (Amp^r^)	7690; 7693	*Kpn*I; *BamH*I; *Xba*I	This study
pSVA5433	pSVA5417‐based expression vector for homologous expression of *dacZ*(D^194^A) with *N*‐terminal 6x His‐Tag in *H. volcanii *(Amp^r^)	9804; 9805	–	This study

**Table A3:** Primers used in this study

Primer number/name	Sequence 5ʹ→3ʹ	Description
pPCR primers		
7687	GAACTGCAGGGGCAACTCCACGAAC	Forward qPCR‐primer amplifying short fragment of *dacZ* (amplicon size: 160 bp)
7688	ATTATTGGGCGACCTCGTGGCGGAC	Reverse qPCR‐primer amplifying short fragment of *dacZ* (amplicon size: 160 bp)
Generation and identification of *Haloferax volcanii* knock‐out strains		
8001	GATAGGTACCCGTACTCCCACTTCAACTTC	Forward primer amplifying upstream flank of *dacZ*/predicted Operon *dacZ+mscS2* for knock‐out construct with *Kpn*I site (cloned in pTA131)
7608	GATTGGATCCTGGTGGAGAGAGACACCGGGTGAG	Reverse primer amplifying upstream flank of *dacZ*/predicted Operon *dacZ+mscS2* for knock‐out construct with *BamH*I site (cloned in pTA131)
7609	TAGTATGGATCCGATGCCGGGGTTCGCTTC	Forward primer amplifying downstream flank of *dacZ* for knock‐out construct with *BamH*I site (cloned in pTA131)
8004	GTACTCTAGACGACGAGCAGTTCGACCTTG	Reverse primer amplifying downstream flank of *dacZ* for knock‐out construct with *Xba*I site (cloned in pTA131)
7610	GCCCTTCTGCCCTTTCTTGC	Forward primer amplifying 113 bp probe of *dacZ*
7634	AGAACATGCGGTCGGGCATC	Reverse primer amplifying 113 bp probe of *dacZ*
7612	TGTTCACGCCCGAGATACTG	Forward primer used to check if knock‐out of *dacZ*/predicted Operon *dacZ+mscS2* was successful (annealing outside the flanking regions used on the knock‐out construct)
7613	AGCGTGGCGAGAAAGAAGAC	Reverse primer used to check if knock‐out of *dacZ* was successful (annealing outside the flanking regions used on the knock‐out construct)
7671	ATATAATGGATCCGCGCTCGGACCCGGGGTCACTG	Forward primer amplifying downstream flank of predicted Operon *dacZ+mscS2* for knock‐out construct with *BamH*I site (cloned in pTA131)
7672	TGCATCTAGAGCCGACACGACCGAATCCAGCACCAC	Reverse primer amplifying downstream flank of predicted Operon *dacZ+mscS2* for knock‐out construct with *Xba*I site (cloned in pTA131)
7683	GAACCGACCGACGAGTGGATGAG	Forward primer amplifying 108 bp probe of predicted Operon *dacZ+mscS2*
7684	CTCGTCCGGGCGTTCAAGG	Reverse primer amplifying 108 bp probe of predicted Operon *dacZ+mscS2*
7673	ATCGACCACATGGCCGAACTC	Reverse primer used to check if knock‐out of predicted Operon *dacZ+mscS2*/*mscS2* was successful (annealing outside the flanking regions used on the knock‐out construct)
7690	GTAGGTACCGCGTGATGCTCAACTACGTGTTCG	Forward primer amplifying upstream flank of *mscS2* for knock‐out construct with *Kpn*I site (cloned in pTA131)
7691	TATTATAGGATCCGCGCTCGGACCCGGGGTCAC	Reverse primer amplifying upstream flank of *mscS2* for knock‐out construct with *BamH*I site (cloned in pTA131)
7692	AATGGATCCCTCAGTACGCCTCCGGGTCGATTTC	Forward primer amplifying downstream flank of *mscS2* for knock‐out construct with *BamH*I site (cloned in pTA131)
7693	TCATTCTAGACGCGTCCGCGTCGAGGAGAACATGC	Reverse primer amplifying downstream flank of *mscS2* for knock‐out construct with *Xba*I site (cloned in pTA131)
7694	CCAGAATGGCGATGAACAAC	Forward primer amplifying 118 bp probe of *mscS2*
7695	TATTTCGTCCTCGGCATCAG	Reverse primer amplifying 118 bp probe of *mscS2*
7611	AACGTCAAAGACCGCATCCGCTTCG	Forward primer used to check if knock‐out of *mscS2* was successful (annealing outside the flanking regions used on the knock‐out construct)
pTA131 fw seq	GGGGGATGTGCTGCAAGG	Forward primer used for sequencing pTA131/pTA356/pTA409/pTA1992 based constructs
pTA131 rv seq	TGACCATGATTACGCCAAGC	Reverse primer used for sequencing pTA131/pTA356/pTA409/pTA1992 based constructs
Generation and identification of *H. volcanii* strains with exchanged promotor		
7654	ATCGCATATGGCGGCGTTATCCGAATTATTGGGCGAC	Forward primer amplifying *dacZ* for generation of tryptophan‐inducible alleles with *Nde*I site (cloned in pTA1369)
7662	ATGCGAATTCTCAGTACGCCTCCGGGTCGATTTCGAG	Reverse primer amplifying *dacZ* for generation of tryptophan‐inducible alleles with *EcoR*I site (cloned in pTA1369)
7610	GCCCTTCTGCCCTTTCTTGC	Forward primer used to check correct (=upstream) pop‐in of tryptophan‐inducible alleles of *dacZ*
7665	TCACGCCCGAGATACTGACAAC	Reverse primer used to check correct (=upstream) pop‐in of tryptophan‐inducible alleles of *dacZ*
Cloning of protein expression constructs		
7669	GATACCATGGCGGCGTTATCCGAATTATTGGG	Forward primer amplifying *dacZ* for homologous protein expression with *Nco*I site (cloned in pTA1992)
7633	GATTGAATTCTCAGTACGCCTCCGGGTCGAT	Reverse primer amplifying *dacZ* for homologous protein expression with *EcoR*I site (cloned in pTA1992)
7644	GCACATGCTCTTCTAGTGCTGCGTTATCCGAATTATTGGGCGAC	Forward primer amplifying *dacZ* for heterologous protein expression with *Sap*I site (cloned with FX‐system in p7XNH3)
7645	GCTGTAGCTCTTCATGCGTACGCCTCCGGGTCGATTTCGAGAAT	Reverse primer amplifying *dacZ* for heterologous protein expression with *Sap*I site (cloned with FX‐system in p7XNH3)
9804	GGCTCGCCGGCGCGTTCGTCATCTC	Froward primer introducing single base pair mutation in pSVA5417 changing codon for Asp^194^ (GAC) to Ala (GCC)
9805	GCGCCGGCGAGCCGCGAGAACTC	Reverse primer introducing single base pair mutation in pSVA5417 changing codon for Asp^194^ (GAC) to Ala (GCC)
p.synF	CGAGAATCGAAACGCTTATAAGTGCCCCCCGGCTAGAGAGAT	Forward primer to generate p.*syn *synthetic promoter (Large et al., 2007)
p.synR2	TAATCTCTCTAGCCGGGGGGCACTTATAAGCGTTTCGATTCT	Reverse primer to generate p.*syn *synthetic promoter (Large et al., 2007)
Genomic *dacZ* knock‐out and *in trans* complementation		
7680	TAAGGTACCGACGGAGCGTAATCGGTCGTCGTAG	Forward primer amplifying *dacZ* +approx. 1,000 bp upstream and downstream as episomal copy with *Kpn*I site (cloned in pTA356/pTA409)
7681	TATGGATCCTGTGGTGTAGACCTTCGCAGGTTCG	Reverse primer amplifying *dacZ* +approx. 1,000 bp upstream and downstream as episomal copy with *BamH*I site (cloned in pTA356/pTA409)
7682	CGACTACGAGTCCTCGCTCGAATACG	Forward primer binding in *trpA* gene to check for correct orientation in *trpA*‐marked deletion construct
8004	GTACTCTAGACGACGAGCAGTTCGACCTTG	Reverse primer binding in downstream flanking region of *dacZ* to check for correct *trpA* orientation in *trpA*‐marked deletion construct
7689	TATCGTTGCGGGAAATGTGC	Forward primer used to check for *dacZ* replacement by *trpA *(annealing outside the upstream regions used on the episomal plasmid)
7673	ATCGACCACATGGCCGAACTC	Reverse primer used to check for *dacZ* replacement by *trpA *(annealing outside the downstream regions used on the episomal plasmid)
9802	AGTTCCGGCCAGAACACGTC	Forward primer amplifying 154 bp probe of *HVO_1658 *for verification of genomic *dacZ* replacement by genomic DNA digestion
9803	GGCGCGTGATGCTCAACTAC	Reverse primer amplifying 154 bp probe of *HVO_1658 *for verification of genomic *dacZ* replacement by genomic DNA digestion

Note

qPCR: quantitative PCR.

## References

[mbo3829-bib-0001] Allers, T. , Ngo, H.‐P. , Mevarech, M. , & Lloyd, R. G. (2004). Development of additional selectable markers for the halophilic archaeon *Haloferax volcanii* based on the leuB and trpA genes. Applied and Environment Microbiology, 70, 943–953. 10.1128/AEM.70.2.943-953.2004 PMC348920 14766575

[mbo3829-bib-0002] Bai, Y. , Yang, J. , Zarrella, T. M. , Zhang, Y. , Metzger, D. W. , & Bai, G. (2014). Cyclic di‐AMP impairs potassium uptake mediated by a cyclic di‐AMP binding protein in Streptococcus pneumoniae. Journal of Bacteriology, 196, 614–623. 10.1128/JB.01041-13 24272783 PMC3911161

[mbo3829-bib-0003] Bai, Y. , Yang, J. , Zhou, X. , Ding, X. , Eisele, L. E. , & Bai, G. (2012). *Mycobacterium tuberculosis* Rv3586 (DacA) is a diadenylate cyclase that converts ATP or ADP into c‐di‐AMP. PLoS ONE, 7, e35206 10.1371/journal.pone.0035206 22529992 PMC3328451

[mbo3829-bib-0004] Baumann, A. , Lange, C. , & Soppa, J. (2007). Transcriptome changes and cAMP oscillations in an archaeal cell cycle. BMC Cell Biology, 8, 21 10.1186/1471-2121-8-21 17562013 PMC1906763

[mbo3829-bib-0005] Bejerano‐Sagie, M. , Oppenheimer‐Shaanan, Y. , Berlatzky, I. , Rouvinski, A. , Meyerovich, M. , & Ben‐Yehuda, S. (2006). A checkpoint protein that scans the chromosome for damage at the start of sporulation in *Bacillus subtilis* . Cell, 125, 679–690. 10.1016/j.cell.2006.03.039 16713562

[mbo3829-bib-0006] Blötz, C. , Treffon, K. , Kaever, V. , Schwede, F. , Hammer, E. , & Stülke, J. (2017). Identification of the components involved in cyclic di‐AMP signaling in *Mycoplasma pneumoniae* . Frontiers in Microbiology, 8, 1328 10.3389/fmicb.2017.01328 28751888 PMC5508000

[mbo3829-bib-0007] Booth, I. R. (2014). Bacterial mechanosensitive channels: Progress towards an understanding of their roles in cell physiology. Current Opinion in Microbiology, 18, 16–22. 10.1016/j.mib.2014.01.005 24607989 PMC4005912

[mbo3829-bib-0008] Bowman, L. , Zeden, M. S. , Schuster, C. F. , Kaever, V. , & Gründling, A. (2016). New insights into the cyclic di‐adenosine monophosphate (c‐di‐AMP) degradation pathway and the requirement of the cyclic dinucleotide for acid stress resistance in *Staphylococcus aureus* . Journal of Biological Chemistry, 291, 26970–26986. 10.1074/jbc.m116.747709 27834680 PMC5207132

[mbo3829-bib-0009] Brendel, J. , Stoll, B. , Lange, S. J. , Sharma, K. , Lenz, C. , Stachler, A.‐E. , … Marchfelder, A. (2014). A complex of Cas proteins 5, 6, and 7 is required for the biogenesis and stability of clustered regularly interspaced short palindromic repeats (crispr)‐derived rnas (crrnas) in *Haloferax volcanii* . Journal of Biological Chemistry, 289, 7164–7177. 10.1074/jbc.M113.508184 24459147 PMC3945376

[mbo3829-bib-0010] Chaudhuri, R. R. , Allen, A. G. , Owen, P. J. , Shalom, G. , Stone, K. , Harrison, M. , … Charles, I. G. (2009). Comprehensive identification of essential *Staphylococcus aureus* genes using Transposon‐Mediated Differential Hybridisation (TMDH). BMC Genomics, 10, 291 10.1186/1471-2164-10-291 19570206 PMC2721850

[mbo3829-bib-0011] Commichau, F. M. , Gibhardt, J. , Halbedel, S. , Gundlach, J. , & Stülke, J. (2017). A delicate connection: c‐di‐AMP affects cell integrity by controlling osmolyte transport. Trends in Microbiology, 26, 175–185. 10.1016/j.tim.2017.09.003 28965724

[mbo3829-bib-0012] Commichau, F. M. , Heidemann, J. L. , Ficner, R. , & Stülke, J. (2018). Making and breaking of an essential poison: The cyclases and phosphodiesterases that produce and degrade the essential second messenger cyclic di‐AMP in bacteria. Journal of Bacteriology, 201 10.1128/JB.00462-18 PMC6287462 30224435

[mbo3829-bib-0013] Commichau, F. M. , & Stülke, J. (2018). Coping with an essential poison: A genetic suppressor analysis corroborates a key function of c‐di‐AMP in controlling potassium ion homeostasis in gram‐positive bacteria. Journal of Bacteriology, 200 10.1128/JB.00166-18 PMC5971470 29610213

[mbo3829-bib-0014] Corrigan, R. M. , Abbott, J. C. , Burhenne, H. , Kaever, V. , & Gründling, A. (2011). c‐di‐AMP is a new second messenger in *Staphylococcus aureus* with a role in controlling cell size and envelope stress. PLoS Path, 7, e1002217 10.1371/journal.ppat.1002217 PMC3164647 21909268

[mbo3829-bib-0015] Corrigan, R. M. , Campeotto, I. , Jeganathan, T. , Roelofs, K. G. , Lee, V. T. , & Gründling, A. (2013). Systematic identification of conserved bacterial c‐di‐AMP receptor proteins. Proceedings of the National Academy of Sciences, 110, 9084–9089. 10.1073/pnas.1300595110 PMC3670340 23671116

[mbo3829-bib-0016] Corrigan, R. M. , & Gründling, A. (2013). Cyclic di‐AMP: Another second messenger enters the fray. Nature Reviews Microbiology, 11, 513–524. 10.1038/nrmicro3069 23812326

[mbo3829-bib-0017] Delmas, S. , Shunburne, L. , Ngo, H.‐P. , & Allers, T. (2009). Mre11‐Rad50 promotes rapid repair of DNA damage in the polyploid archaeon *Haloferax volcanii* by restraining homologous recombination. PLoS Genetics, 5, e1000552 10.1371/journal.pgen.1000552 19593371 PMC2700283

[mbo3829-bib-0018] Ducret, A. , Quardokus, E. M. , & Brun, Y. V. (2016). MicrobeJ, a tool for high throughput bacterial cell detection and quantitative analysis. Nature Microbiology, 1, 16077 10.1038/nmicrobiol.2016.77 PMC5010025 27572972

[mbo3829-bib-0019] Duggin, I. G. , Aylett, C. H. S. , Walsh, J. C. , Michie, K. A. , Wang, Q. , Turnbull, L. , … Löwe, J. (2015). CetZ tubulin‐like proteins control archaeal cell shape. Nature, 519, 362–365. 10.1038/nature13983 25533961 PMC4369195

[mbo3829-bib-0020] Edwards, M. D. , Black, S. , Rasmussen, T. , Rasmussen, A. , Stokes, N. R. , Stephen, T.‐L. , … Booth, I. R. (2012). Characterization of three novel mechanosensitive channel activities in *Escherichia coli* . Channels (Austin), 6, 272–281. 10.4161/chan.20998 22874652 PMC3508906

[mbo3829-bib-0021] Fahmi, T. , Port, G. C. , & Cho, K. H. (2017). c‐di‐AMP: An essential molecule in the signaling pathways that regulate the viability and virulence of gram‐positive bacteria. Genes (Basel), 8, 1–17. 10.3390/genes8080197 PMC5575661 28783096

[mbo3829-bib-0022] Felsenstein, J. (1985). Confidence limits on phylogenies: An approach using the bootstrap. Evolution, 39, 783–791. 10.1111/j.1558-5646.1985.tb00420.x 28561359

[mbo3829-bib-0023] Gamble‐Milner, R. J. (2016 ). Genetic analysis of the Hel308 helicase in the archaeon *Haloferax volcanii* . PhD thesis, University of Nottingham. Retrieved from http://eprints.nottingham.ac.uk/37153/1/Rebecca%20Gamble-Milner.pdf

[mbo3829-bib-0024] Geertsma, E. R. , & Dutzler, R. (2011). A versatile and efficient high‐throughput cloning tool for structural biology. Biochemistry, 50, 3272–3278. 10.1021/bi200178z 21410291

[mbo3829-bib-0025] Gomelsky, M. (2011). cAMP, c‐di‐GMP, c‐di‐AMP and now cGMP: Bacteria use them all!. Molecular Microbiology, 79, 562–565. 10.1111/j.1365-2958.2010.07514.x 21255104 PMC3079424

[mbo3829-bib-0026] Gundlach, J. , Herzberg, C. , Kaever, V. , Gunka, K. , Hoffmann, T. , Weiß, M. , … Stülke, J. (2017). Control of potassium homeostasis is an essential function of the second messenger cyclic di‐AMP in *Bacillus subtilis* . Science Signalling, 10, eaal3011 10.1126/scisignal.aal3011 28420751

[mbo3829-bib-0027] Gundlach, J. , Mehne, F. M. P. , Herzberg, C. , Kampf, J. , Valerius, O. , Kaever, V. , & Stülke, J. (2015). An essential poison: Synthesis and degradation of cyclic di‐AMP in *Bacillus subtilis* . Journal of Bacteriology, 197, 3265–3274. 10.1128/JB.00564-15 26240071 PMC4573722

[mbo3829-bib-0028] Hartman, A. L. , Norais, C. , Badger, J. H. , Delmas, S. , Haldenby, S. , Madupu, R. , … Eisen, J. A. (2010). The complete genome sequence of *Haloferax volcanii* DS2, a model archaeon. PLoS ONE, 5, e9605 10.1371/journal.pone.0009605 20333302 PMC2841640

[mbo3829-bib-0029] Huynh, T. N. , & Woodward, J. J. (2016). Too much of a good thing: Regulated depletion of c‐di‐AMP in the bacterial cytoplasm. Current Opinion in Microbiology, 30, 22–29. 10.1016/j.mib.2015.12.007 26773214 PMC4821758

[mbo3829-bib-0030] JCSG . Crystal structure of hypothetical protein (NP_068944.1) from *Archaeoglobus fulgidus* at 1.30 A resolution.

[mbo3829-bib-0031] Jones, D. T. , Taylor, W. R. , & Thornton, J. M. (1992). The rapid generation of mutation data matrices from protein sequences. Computer Applications in the Biosciences, 8, 275–282. 10.1093/bioinformatics/8.3.275 1633570

[mbo3829-bib-0032] Kalia, D. , Merey, G. , Nakayama, S. , Zheng, Y. , Zhou, J. , Luo, Y. , … Sintim, H. O. (2013). Nucleotide, c‐di‐GMP, c‐di‐AMP, cGMP, cAMP, (p)ppGpp signaling in bacteria and implications in pathogenesis. Chemical Society Reviews, 42, 305–341. 10.1039/C2CS35206K 23023210

[mbo3829-bib-0033] Kellenberger, C. A. , Chen, C. , Whiteley, A. T. , Portnoy, D. A. , & Hammond, M. C. (2015). RNA‐based fluorescent biosensors for live cell imaging of second messenger cyclic di‐AMP. Journal of the American Chemical Society, 137, 6432–6435. 10.1021/jacs.5b00275 25965978 PMC4521591

[mbo3829-bib-0034] Kumar, S. , Stecher, G. , & Tamura, K. (2016). MEGA7: Molecular evolutionary genetics analysis version 7.0 for bigger datasets. Molecular Biology and Evolution, 33, 1870–1874. 10.1093/molbev/msw054 27004904 PMC8210823

[mbo3829-bib-0035] Large, A. , Stamme, C. , Lange, C. , Duan, Z. , Allers, T. , Soppa, J. , & Lund, P. A. (2007). Characterization of a tightly controlled promoter of the halophilic archaeon *Haloferax volcanii* and its use in the analysis of the essential cct1 gene. Molecular Microbiology, 66, 1092–1106. 10.1111/j.1365-2958.2007.05980.x 17973910

[mbo3829-bib-0036] Leichtling, B. H. , Rickenberg, H. V. , Seely, R. J. , Fahrney, D. E. , & Pace, N. R. (1986). The occurrence of cyclic AMP in archaebacteria. Biochemical and Biophysical Research Communications, 136, 1078–1082. 10.1016/0006-291X(86)90443-2 3013165

[mbo3829-bib-0037] Lestini, R. , Duan, Z. , & Allers, T. (2010). The archaeal Xpf/Mus81/FANCM homolog Hef and the Holliday junction resolvase Hjc define alternative pathways that are essential for cell viability in *Haloferax volcanii* . DNA Repair (Amst), 9, 994–1002. 10.1016/j.dnarep.2010.06.012 20667794

[mbo3829-bib-0038] Lucht, J. M. , & Bremer, E. (1994). Adaptation of *Escherichia coli* to high osmolarity environments: Osmoregulation of the high‐affinity glycine betaine transport system proU. FEMS Microbiology Reviews, 14, 3–20. 10.1016/0168-6445(94)90008-6 8011357

[mbo3829-bib-0039] Luo, Y. , & Helmann, J. D. (2012). Analysis of the role of *Bacillus subtilis* σ(M) in β‐lactam resistance reveals an essential role for c‐di‐AMP in peptidoglycan homeostasis. Molecular Microbiology, 83, 623–639. 10.1111/j.1365-2958.2011.07953.x 22211522 PMC3306796

[mbo3829-bib-0040] Manikandan, K. , Sabareesh, V. , Singh, N. , Saigal, K. , Mechold, U. , & Sinha, K. M. (2014). Two‐step synthesis and hydrolysis of cyclic di‐AMP in Mycobacterium tuberculosis. PLoS ONE, 9, e86096 10.1371/journal.pone.0086096 24465894 PMC3900455

[mbo3829-bib-0041] Marchler‐Bauer, A. , Bo, Y. , Han, L. , He, J. , Lanczycki, C. J. , Lu, S. , … Bryant, S. H. (2017). CDD/SPARCLE: Functional classification of proteins via subfamily domain architectures. Nucleic Acids Research, 45(D1), D200–D203. 10.1093/nar/gkw1129 27899674 PMC5210587

[mbo3829-bib-0042] McDonough, K. A. , & Rodriguez, A. (2011). The myriad roles of cyclic AMP in microbial pathogens: From signal to sword. Nature Reviews Microbiology, 10, 27–38. 10.1038/nrmicro2688 22080930 PMC3785115

[mbo3829-bib-0043] McLaggan, D. , Naprstek, J. , Buurman, E. T. , & Epstein, W. (1994). Interdependence of K+ and glutamate accumulation during osmotic adaptation of *Escherichia coli* . Journal of Biological Chemistry, 269, 1911–1917. 7904996

[mbo3829-bib-0044] Mehne, F. M. P. , Gunka, K. , Eilers, H. , Herzberg, C. , Kaever, V. , & Stülke, J. (2013). Cyclic di‐AMP homeostasis in *Bacillus subtilis*: Both lack and high level accumulation of the nucleotide are detrimental for cell growth. Journal of Biological Chemistry, 288, 2004–2017. 10.1074/jbc.M112.395491 23192352 PMC3548507

[mbo3829-bib-0045] Mehne, F. M. P. , Schröder‐Tittmann, K. , Eijlander, R. T. , Herzberg, C. , Hewitt, L. , Kaever, V. , et al. (2014). Control of the diadenylate cyclase CdaS in *Bacillus subtilis*: An autoinhibitory domain limits cyclic di‐AMP production. Journal of Biological Chemistry, 289, 21098–21107. 10.1074/jbc.M114.562066 24939848 PMC4110313

[mbo3829-bib-0046] Meury, J. , & Kohiyama, M. (1989). ATP is required for K+ active transport in the archaebacterium *Haloferax volcanii* . Archives of Microbiology, 151, 530–536. 10.1007/BF00454870

[mbo3829-bib-0047] Miller, J. H. (1971). Experiments in molecular genetics. Cold Spring Harbor, NY: Cold Spring Harbor Laboratory.

[mbo3829-bib-0048] Norais, C. , Hawkins, M. , Hartman, A. L. , Eisen, J. A. , Myllykallio, H. , & Allers, T. (2007). Genetic and physical mapping of DNA replication origins in *Haloferax volcanii* . PLoS Genetics, 3, e77 10.1371/journal.pgen.0030077 17511521 PMC1868953

[mbo3829-bib-0049] Oren, A. (2008). Microbial life at high salt concentrations: Phylogenetic and metabolic diversity. Saline Systems, 4, 2 10.1186/1746-1448-4-2 18412960 PMC2329653

[mbo3829-bib-0050] Römling, U. (2008). Great times for small molecules: c‐di‐AMP, a second messenger candidate in Bacteria and Archaea. Science Signaling, 1, pe39 10.1126/scisignal.133pe39 18714086

[mbo3829-bib-0051] Rosenberg, J. , Dickmanns, A. , Neumann, P. , Gunka, K. , Arens, J. , Kaever, V. , … Commichau, F. M. (2015). Structural and biochemical analysis of the essential diadenylate cyclase CdaA from *Listeria monocytogenes* . Journal of Biological Chemistry, 290, 6596–6606. 10.1074/jbc.M114.630418 25605729 PMC4358292

[mbo3829-bib-0052] Rouillon, C. , Athukoralage, J. S. , Graham, S. , Grüschow, S. , & White, M. F. (2018). Control of cyclic oligoadenylate synthesis in a type III CRISPR system. Elife, 7, 1–25. 10.7554/eLife.36734 PMC6053304 29963983

[mbo3829-bib-0053] Schindelin, J. , Arganda‐Carreras, I. , Frise, E. , Kaynig, V. , Longair, M. , Pietzsch, T. , … Cardona, A. (2012). Fiji: An open‐source platform for biological‐image analysis. Nature Methods, 9, 676–682. 10.1038/nmeth.2019 22743772 PMC3855844

[mbo3829-bib-0054] Shenroy, A. R. , & Visweswariah, S. S. (2004). Class III nucleotide cyclases in bacteria and archaebacteria: Lineage‐specific expansion of adenylyl cyclases and a dearth of guanylyl cyclases. FEBS Letters, 561, 11–21. 10.1016/S0014-5793(04)00128-0 15043055

[mbo3829-bib-0055] Skowyra, A. , & MacNeill, S. A. (2012). Identification of essential and non‐essential single‐stranded DNA‐binding proteins in a model archaeal organism. Nucleic Acids Research, 40, 1077–1090. 10.1093/nar/gkr838 21976728 PMC3273820

[mbo3829-bib-0056] Song, J.‐H. , Ko, K. S. , Lee, J.‐Y. , Baek, J. Y. , Oh, W. S. , Yoon, H. S. , … Chun, J. (2005). Identification of essential genes in *Streptococcus pneumoniae* by allelic replacement mutagenesis. Molecules and Cells, 19, 365–374. 15995353

[mbo3829-bib-0057] Spangler, C. , Böhm, A. , Jenal, U. , Seifert, R. , & Kaever, V. (2010). A liquid chromatography‐coupled tandem mass spectrometry method for quantitation of cyclic di‐guanosine monophosphate. Journal of Microbiol Methods, 81, 226–231. 10.1016/j.mimet.2010.03.020 20385176

[mbo3829-bib-0058] Stroud, A. , Liddell, S. , & Allers, T. (2012). Genetic and biochemical identification of a novel single‐stranded DNA‐binding complex in *Haloferax volcanii* . Frontiers in Microbiology, 3, 224 10.3389/fmicb.2012.00224 22719738 PMC3376784

[mbo3829-bib-0059] Sutherland, E. W. , & Wosilait, W. D. (1956). The relationship of epinephrine and glucagon to liver phosphorylase. I. Liver phosphorylase; preparation and properties. Journal of Biological Chemistry, 218, 459–468. 13278353

[mbo3829-bib-0060] Thombre, R. S. , Shinde, V. D. , Oke, R. S. , Dhar, S. K. , & Shouche, Y. S. (2016). Biology and survival of extremely halophilic archaeon Haloarcula marismortui RR12 isolated from Mumbai salterns, India in response to salinity stress. Scientific Reports, 6, 25642 10.1038/srep25642 27231230 PMC4882750

[mbo3829-bib-0061] Waterhouse, A. , Bertoni, M. , Bienert, S. , Studer, G. , Tauriello, G. , Gumienny, R. , … Schwede, T. (2018). SWISS‐MODEL: Homology modelling of protein structures and complexes. Nucleic Acids Research, 46(W1), W296–W303. 10.1093/nar/gky427 29788355 PMC6030848

[mbo3829-bib-0062] Whiteley, A. T. , Pollock, A. J. , & Portnoy, D. A. (2015). The PAMP c‐di‐AMP is essential for *Listeria monocytogenes* growth in rich but not minimal media due to a toxic increase in (p)ppGpp. [corrected]. Cell Host & Microbe, 17, 788–798. 10.1016/j.chom.2015.05.006 26028365 PMC4469362

[mbo3829-bib-0063] Witte, G. , Hartung, S. , Büttner, K. , & Hopfner, K.‐P. (2008). Structural biochemistry of a bacterial checkpoint protein reveals diadenylate cyclase activity regulated by DNA recombination intermediates. Molecular Cell, 30, 167–178. 10.1016/j.molcel.2008.02.020 18439896

[mbo3829-bib-0064] Woodward, J. J. , Iavarone, A. T. , & Portnoy, D. A. (2010). c‐di‐AMP secreted by intracellular *Listeria monocytogenes* activates a host type I interferon response. Science, 328, 1703–1705. 10.1126/science.1189801 20508090 PMC3156580

[mbo3829-bib-0065] Yang, J. , Bai, Y. , Zhang, Y. , Gabrielle, V. D. , Jin, L. , & Bai, G. (2014). Deletion of the cyclic di‐AMP phosphodiesterase gene (cnpB) in *Mycobacterium tuberculosis* leads to reduced virulence in a mouse model of infection. Molecular Microbiology, 93, 65–79. 10.1111/mmi.12641 24806618 PMC4088933

[mbo3829-bib-0066] Zhang, L. , Li, W. , & He, Z. G. (2013). DarR, a TetR‐like transcriptional factor, is a cyclic di‐AMP‐responsive repressor in *Mycobacterium smegmatis* . Journal of Biological Chemistry, 288, 3085–3096. 10.1074/jbc.M112.428110 23250743 PMC3561532

